# Prevalence, Characteristics, and Clonal Distribution of *Escherichia coli* Carrying Mobilized Colistin Resistance Gene *mcr-1.1* in Swine Farms and Their Differences According to Swine Production Stages

**DOI:** 10.3389/fmicb.2022.873856

**Published:** 2022-05-06

**Authors:** Soomin Lee, Jae-Uk An, JungHa Woo, Hyokeun Song, Saehah Yi, Woo-Hyun Kim, Ju-Hoon Lee, Sangryeol Ryu, Seongbeom Cho

**Affiliations:** ^1^College of Veterinary Medicine and Research Institute for Veterinary Science, Seoul National University, Seoul, South Korea; ^2^Department of Food and Animal Biotechnology, Seoul National University, Seoul, South Korea; ^3^Department of Agricultural Biotechnology, Center for Food Bioconvergence, Seoul National University, Seoul, South Korea

**Keywords:** colistin, *mcr-1.1*, intestinal pathogenic *E. coli*, extra-intestinal pathogenic *E. coli*, extended-spectrum β-lactamase, swine production stages

## Abstract

Global spread of *Escherichia coli* strains carrying the mobilized colistin resistance gene *mcr-1.1* (MCR1-EC) poses serious threats to public health. Colistin has been generally prescribed for swine colibacillosis, having made swine farms as major reservoirs of MCR1-EC. The present study aimed to understand characteristic differences of MCR1-EC, including prevalence, antimicrobial resistance, and virulence, according to swine production stages. In addition, genetic relatedness was evaluated between MCR1-EC isolated from this study as well as pig-, human-, and chicken-derived strains published in the National Center for Biotechnology Information (NCBI), based on the multi-locus sequence types (MLSTs) and whole-genome sequences (WGS). Individual fecal samples (*n* = 331) were collected from asymptomatic weaning-piglets, growers, finishers, and sows from 10 farrow-to-finishing farms in South Korea between 2017 and 2019. The weighted prevalence of MCR1-EC was 11.6% (95% CI: 8.9%–15.0%, 55/331), with the highest prevalence at weaning stage. The 96.2% of MCR1-EC showed multi-drug resistance. Notably, weaning stage-derived MCR1-EC showed higher resistance rates (e.g., against extended-spectrum β-lactams or quinolones) than those from other stages. MCR1-EC with virulence advantages (e.g., intestinal/extraintestinal pathogenic *E. coli* or robust biofilm formation) were identified from all pig stages, accounting for nearly half of the total strains. WGS-based in-depth characterization showed that intestinal pathogenic MCR1-EC harbored multi-drug resistance and multiple virulence factors, which were highly shared between strains isolated from pigs of different stages. The clonal distribution of MCR1-EC was shared within swine farms but rarely across farms. The major clonal type of MCR1-EC from swine farms and NCBI database was ST10-A. Core genomes of MCR1-EC isolated from individuals within closed environments (same farms or human hospitals) were highly shared (genetic distance < 0.01), suggesting a high probability of clonal expansion of MCR1-EC within closed environments such as livestock husbandry. To the best of our knowledge, this is the first study to analyze the differences in the characteristics and clonal distribution of MCR1-EC according to production stages in swine farms, an important reservoir of MCR1-EC. Our results highlight the need to establish MCR1-EC control plans in swine farms based on an in-depth understanding of MCR1-EC characteristics according to swine production stages, focusing especially on the weaning stages.

## Introduction

Colistin is regarded as a last resort for the treatment of multi-drug resistant (MDR) bacterial infections in humans and has been classified as a critically important antimicrobial agent by the World Health Organization (WHO, 2018). Before 2016, colistin resistance was mainly considered to be associated with mutational and regulatory changes in chromosomal genes, including *pmrAB* and *phoPQ* (Liu et al., 2016). The mobilized colistin resistance gene *mcr-1* was first described in a plasmid carried by *Escherichia coli* strains in 2016 (Liu et al., 2016), and has since been found in more than 50 countries across six continents (Wang et al., 2018b), highlighting the global spread of colistin resistance *via mcr-1*.

Swine colibacillosis is a major disease in pigs that causes huge economic losses for the global swine industry (Luppi, 2017). Colistin has been generally used for the treatment of swine colibacillosis, leading to an increased prevalence of *E. coli* strains carrying *mcr-1* (MCR1-EC) in swine farms (Malhotra-Kumar et al., 2016; Tong et al., 2018; Liu et al., 2020; Nakano et al., 2021). In pig production systems, pigs at different stages of growth, referred to as weaning piglets, growers, finishers, and pregnant pigs, are usually raised in separate barns (Kyriazakis, 2006). However, as pigs age and transition to the next growth stage and next stage barn, bacterial transmission can occur between animals at different swine production stages within farms, which has been reported to be a significant risk factor for the high prevalence of MDR bacteria in swine farms (Fromm et al., 2014; Schmithausen et al., 2015). Since *mcr-1* is mainly mediated by plasmids, the important role of genetic transferability of *mcr-1* in the spread of MCR1-EC has been continuously highlighted in various studies (Garcia et al., 2018; Wang et al., 2018b; Wu et al., 2018b; Soliman et al., 2021). However, genetic transfer essentially presupposes the transfer of strains and bacteria-to-bacteria interactions under favorable conditions (e.g., physical distance between strains, nutrition, and environmental conditions, etc.; Virolle et al., 2020), which suggests that bacterial transmission also provides a crucial basis for the spread of MCR1-EC. Understanding the genetic characteristics and distribution of MCR1-EC considering swine production stages, which is an important reservoir of MCR1-EC, could be a cornerstone to establish strategies for the control of colistin resistance in the swine industry. However, despite its importance, the characteristics and distribution of MCR1-EC based on different swine production stages within farms have rarely been studied.

Given that colistin has been considered a recommended treatment option for swine colibacillosis and that intestinal pathogenic *E. coli* (InPEC) comprises major causative pathogens of swine colibacillosis (Malhotra-Kumar et al., 2016; Tong et al., 2018; Liu et al., 2020; Nakano et al., 2021), the presence of intestinal pathogenic MCR1-EC in pig husbandry represents a severe challenge for the swine industry. Colistin administration during the treatment of swine colibacillosis caused by intestinal pathogenic MCR1-EC can lead to disease treatment failure, as well as complications, resulting in serious economic losses for pig farms (Garcia et al., 2018). To establish suitable strategies to control intestinal pathogenic MCR1-EC in swine farms, an in-depth characterization of intestinal pathogenic MCR1-EC should be performed, and whole-genome sequence (WGS)-based analysis might provide valuable insights.

The present study aimed to understand the characteristic differences and clonal distribution of MCR1-EC in swine farms according to production stages. For this, first, the prevalence, antimicrobial resistance, and genetic and phenotypic virulence characteristics of MCR1-EC isolated from swine farms were investigated, and differences according to swine production stages were analyzed. Second, we performed WGS for all intestinal pathogenic MCR1-EC isolated in this study and conducted an in-depth genetic characterization. Finally, to understand spread characteristics of MCR1-EC, genetic relatedness analysis based on the clone types and WGS were conducted for MCR1-EC strains isolated in this study, as well as MCR1-EC isolated from various sources published in the National Center for Biotechnology Information (NCBI) GenBank database.

## Materials and Methods

### Sample Collection

**Figure 1:**
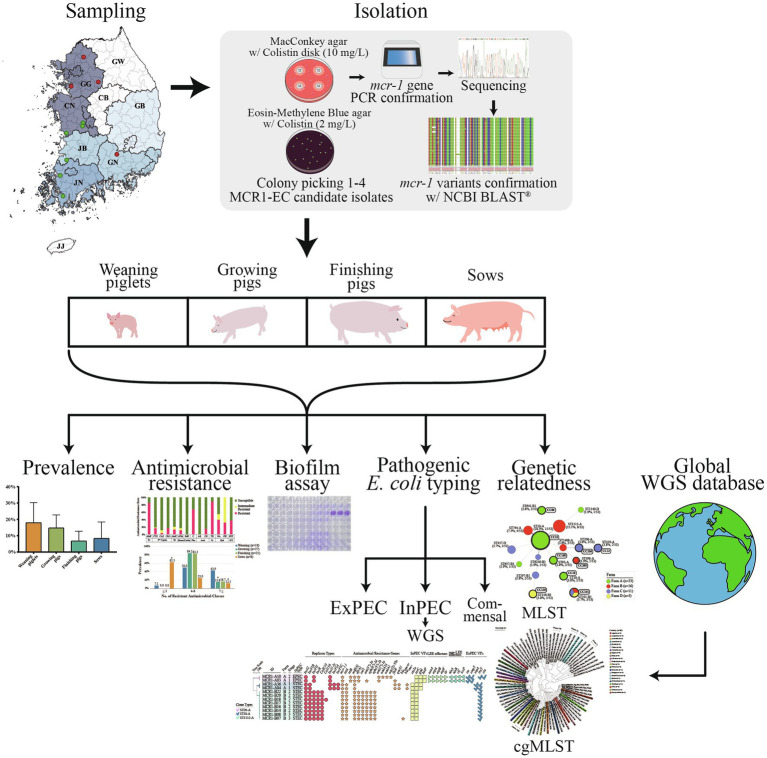
Flow chart of the present study design.

**Figure 2:**
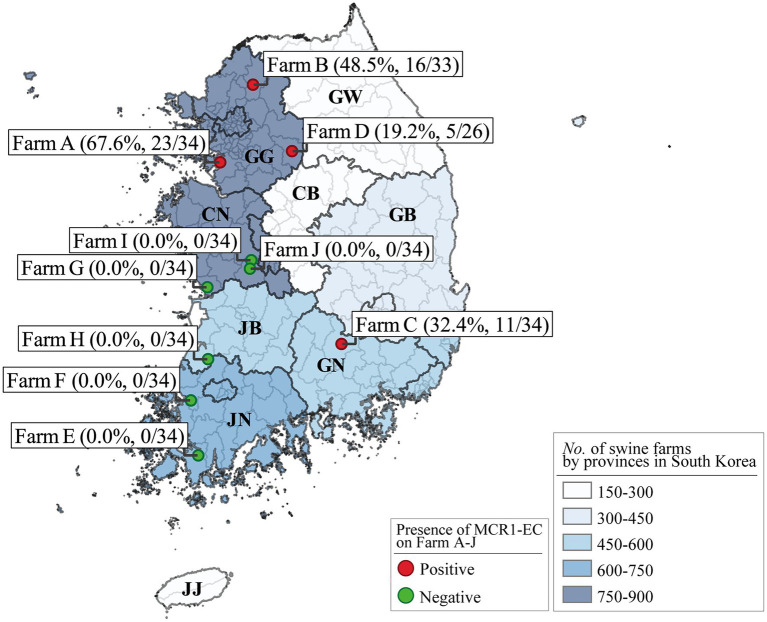
*Escherichia coli* strains carrying *mcr-1* (MCR1-EC) prevalence in Farms A–J and number of swine farms by South Korean province. The numbers in parentheses show the unweighted prevalence of MCR1-EC and the number of MCR1-EC-positive samples versus that of total samples for each farm. The number of pig farms by province in South Korea was obtained from the 2017 demographic report of the Korean Statistical Information Service of Statistics Korea. Visualization was conducted using the QGIS geographic information system program (v3.16.15). MCR1-EC, *Escherichia coli* carrying the mobilized colistin resistance gene *mcr-1.1*; GG, Gyeonggi-do; CN, Chungcheong-nam-do; JB, Jeolla-buk-do; JN, Jeolla-nam-do; GN, Gyeongsang-nam-do; GB, Gyeongsang-buk-do; CB, Chungcheong-buk-do; and GW, Gangwon-do; JJ, Jeju-do.

We collected individual swine fecal samples from 10 “farrow-to-finishing” swine farms located in the five provinces with the highest number of pig farms in South Korea, specifically Gyeonggi-do, Chungcheong-nam-do, Jeolla-nam-do, Jeolla-buk-do, and Gyeongsang-buk-do (Figures 1, 2; Supplementary Table 1). The number of pig farms by province in South Korea was obtained from the 2017 demographic report of the Korean Statistical Information Service of Statistics Korea (Korea, 2017). Each swine farm was visited once between May 2017 and July 2019, and fecal samples were randomly collected from 26 to 34 asymptomatic pigs for each farm, including 5–6 weaning piglets (4–7-week-old), 9–11 growing pigs (7–14-week-old), 8–11 finishing pigs (14–24-week-old), and 3–6 sows, and immediately transported to the lab. In total, 331 fecal samples (59 from weaners, 108 from growers, 107 from finishers, and 57 from pregnant sows) were included.

### Isolation of MCR1-EC

The isolation of MCR1-EC was conducted following previously described protocols for the isolation of antimicrobial resistant *E. coli*, with slight modifications (Bartoloni et al., 1998, 2006; Wedley et al., 2011, 2017). Approximately, 1 g of each sample was resuspended in 9 ml of *Escherichia coli* broth (BD Biosciences, New Jersey, United States) and incubated overnight at 37°C. Thereafter, 100 μl of culture suspension was spread on MacConkey agar (BD Biosciences), and a colistin disk (10 μg/ml, Oxoid, Cheshire, United Kingdom) was placed on the plate. After overnight incubation at 37°C, 1–4 colistin-resistant *E. coli* candidate isolates grown inside the colistin-resistant zone (≤10 mm) were selected and streaked on Eosin Methylene Blue agar (BD Biosciences) containing 2 mg/L colistin (Sigma Aldrich, Massachusetts, United States) for further confirmation. The diameter (≤10 mm) of the candidate colistin-resistant zone was set with reference to the disk diffusion quality control range of *E. coli* reference strain ATCC 25922 described in the Clinical Laboratory Standard Institute (CLSI) guidelines M100S 31th Edition (2021). Then, the presence of *mcr-1* and *mcr-1*-encoded replicon types was determined *via* PCR and sequencing as previously described (Wu et al., 2018b). The sequenced PCR amplicons were compared with the reference sequences from the NCBI GenBank database using the Basic Local Alignment Search Tool (BLAST; https://blast.ncbi.nlm.nih.gov/Blast.cgi) to identify the replicon types, as well as *mcr-1* variants from *mcr-1.1* to *mcr-1.32*. The PCR was performed using a SimpliAmp Thermal Cycler (Thermo Fisher Scientific, Massachusetts, United States), and sequencing was performed using an ABI PRISM 3730XL DNA analyzer (Thermo Fisher Scientific). *Escherichia coli* isolates carrying *mcr-1* were confirmed as MCR1-EC, and one MCR1-EC strain per sample was randomly selected if more than one isolates were identified from a sample. Primer sequences and reaction conditions are summarized in Supplementary Table 2.

### Antimicrobial Resistance

#### Antimicrobial Susceptibility Assay

Minimum inhibitory concentration (MIC) tests were conducted to evaluate colistin resistance using the Trekstar Sensititre KNIHCOL custom panel (colistin test range: 0.25–128 μg/ml, Trek Diagnostic Systems, Ohio, United States) according to the manufacturer’s instructions. Each isolate was tested in duplicate for the MIC of colistin. Kirby-Bauer disk diffusion susceptibility tests (KBTs) were conducted for 11 antimicrobial classes comprising 14 antimicrobial agents using antimicrobial disks from Oxoid (Cheshire, United Kingdom) as follows: ampicillin (10 μg/ml), cefotaxime (30 μg/ml), ceftazidime (30 μg/ml), ceftriaxone (30 μg/ml), amoxicillin/clavulanate (20/10 μg/ml), aztreonam (30 μg/ml), imipenem (10 μg/ml), chloramphenicol (30 μg/ml), amikacin (30 μg/ml), gentamycin (10 μg/ml), tetracycline (30 μg/ml), nalidixic acid (30 μg/ml), ciprofloxacin (5 μg/ml), and sulfamethoxazole/trimethoprim (1.25/23.75 μg/ml). The MIC tests and KBs results were interpreted according to the CLSI guidelines M100S 31th Edition (2021), and the *E. coli* reference strain ATCC 25922 was used for quality control. When the isolate was resistant to at least one antimicrobial agent belonging to the antimicrobial class, we determined that this isolate was resistant to this antimicrobial class. Then, we calculated the average number of antimicrobial classes to which MCR1-EC strains were resistant. Extended-spectrum β-lactamase (ESBL) phenotypes were determined *via* a standard double-disk test according to CLSI guidelines using four antimicrobial disks from BD Bioscience (New Jersey, United States) as follows: cefotaxime (30 μg/ml), ceftazidime (30 μg/ml), cefotaxime/clavulanate (30/10 μg/ml), and ceftazidime/clavulanate (30/10 μg/ml).

#### Antimicrobial Resistance Genes and Replicon Typing

The presence of genes conferring resistance to β-lactams, chloramphenicol, aminoglycoside, quinolones, and sulfonamide/trimethoprim was determined by PCR. The ESBL genotypes were determined by PCR and sequencing as previously described (Jouini et al., 2007). PCR-based replicon typing was conducted as previously described (Carattoli et al., 2005; Johnson et al., 2012; Lv et al., 2013). Primer sequences and reaction conditions are summarized in Supplementary Table 2.

### Classification of Pathogenic *Escherichia coli*

To analyze the genotypic virulence characteristics of MCR1-EC, we investigated the presence of virulence factors associated with InPEC, extra-intestinal pathogenic *E. coli* (ExPEC), and uro-pathogenic *E. coli* (UPEC). The classification of InPEC was conducted by PCR for the following five InPEC types: shiga toxin-producing *E. coli* (STEC) carrying *stx1* or *stx2*, enteropathogenic *E. coli* (EPEC) carrying *eaeA* or *bfpB*, enteroaggregative *E. coli* (EAEC) carrying *aggR*, enteroinvasive *E. coli* (EIEC) carrying *ipaH*, and enterotoxigenic *E. coli* (ETEC) carrying *lt*, *sta*, or *stb*. The carriage of 21 ExPEC-associated virulence factors associated with adhesion (*csgA*, *fimH*, *sfa/focDE*, *afa/draBC*, *papC*, *papAH*, *yfcV*, and *iha*), toxins (*hlyF*, *astA*, *pic*, *vat*, and *aat*), protectin/serum resistance (*traT*, *ompT*, *iss*, and *kpsMTII*), and siderophores (*fyuA*, *iroNE.coli*, *iutA*, and *chuA*) were investigated using PCR. The classification of ExPEC was conducted following the previously described criteria, specifically positive for ≥2 of five key markers as follows: *papA* and/or *papC*, *sfa/focDE*, *afa/draBC*, *iutA*, and *kpsMTII* (Johnson et al., 2003). The classification of UPEC was conducted following previously described criteria, specifically positive for ≥3 of four key markers as follows: *vat*, *fyuA*, *chuA*, and *yfcV* (Spurbeck et al., 2012). Finally, since all pigs included in this study were healthy, without showing any disease symptoms, *E. coli* isolates that not classified as InPEC, ExPEC, or UPEC were then classified as commensal *E. coli* strains. Primer sequences and reaction conditions are summarized in Supplementary Table 3.

### Phenotypic Assay

#### Conjugation Assay

Conjugation assays were conducted to evaluate the horizontal genetic transferability of *mcr-1* with the *E. coli* J53-Azi^R^ strain as the recipient and 53 MCR1-EC strains as the donors. The conjugation assay was conducted following a previously described protocol with modifications (Kim et al., 2019). Briefly, overnight cultures of donor and recipient strains in Luria-Bertani broth were mixed at a ratio of 1:1, followed by incubation at 37°C for 18 h with constant shaking. Then, 100 μl of the mixture of donor and recipient cells were spread on LB agars supplemented with 2 mg/L colistin (Sigma Aldrich, Massachusetts, United States) and 100 mg/L sodium azide (Sigma Aldrich), followed by overnight incubation at 37°C. The presence of *mcr-1* in conjugants was confirmed *via* PCR.

#### Biofilm Assay

To analyze the phenotypic virulence characteristics of MCR1-EC, biofilm production assays were performed following a previously described protocol with modifications (Nandanwar et al., 2014). Briefly, overnight M9 minimal medium [200 ml/L of M9 media (5X, Sigma-Aldrich), 0.4 g/L of glucose (Sigma-Aldrich), 2 ml/L of MgSO_4_ solution (1 M, Sigma-Aldrich), and 100 μl/L of CaCl_2_ solution (1 M, Sigma-Aldrich)] culture was diluted in fresh M9 minimal medium to a McFarland scale of 0.5. Approximately, 100 μl of this dilution was added into a 96-well microtiter plate and incubated for 24 h at 28°C under stationary conditions. Each bacterial suspension was inoculated into three wells of a microtiter plate. Growth optical densities (ODs) were measured at *λ* = 595 nm with a multiplate reader (Bio-Rad, California, United States). The wells were then washed once with 200 μl of phosphate-buffered saline, dried for 20 min, and stained with 100 μl of 1% crystal violet for 1 h. This was followed by gentle washing with 200 μl of distilled water four times and air-drying for 1 h. The absorbed dye was solubilized in 100 μl of absolute ethanol, and ODs were read at 595 nm. The extent of biofilm formation was calculated using the following formula: SBF = (AB−CW)/G, where SBF is the specific biofilm formation index, AB is the OD595 of the stained bacteria, CW is the OD595 of the stained control wells containing absolute media without bacteria, and G is the OD595 corresponding to cell growth in the media. *Escherichia coli* ATCC 25922 was used as the positive control, whereas the culture medium was used as the negative control. The degree of biofilm production was classified into three categories, weak (SBF < 0.5), moderate (0.5 ≤ SBF < 1.0), and strong (SBF ≥ 1.0).

### Genetic Relatedness Analysis and WGS

#### Clonal Distribution Analysis of MCR1-EC Based on Multi-Locus Sequence Typing and *E. coli* Phylogroup Typing

Multi-Locus Sequence Typing (MLST) was performed as previously described (Wirth et al., 2006). A detailed scheme describing gene amplification, allelic type, and sequence type (ST) assignment methods is available on the pubMLST website.^1^ The minimum spanning tree (MST) based on allelic profiles of seven MLST housekeeping genes was constructed using BioNumerics software (v6.6, Applied Maths, Sint-Martens-Latem, Belgium). The PCR-based PG typing was conducted as previously described (Clermont et al., 2013), and primer sequences and reaction conditions are summarized in Supplementary Table 3.

Further, we analyzed the clonal distribution of 1,652 MCR1-EC strains, of which WGS was publicly available in the NCBI database (accessed on 07 Jan 2020, https://www.ncbi.nlm.nih.gov/pathogens/isolates/), including strains isolated from humans (*n* = 940), chickens (*n* = 446), and pigs (*n* = 226). In addition, we also analyzed the clonal distribution of 17 South Korean-derived MCR1-EC strains, of which WGS was available in the NCBI database, including strains isolated from humans (*n* = 13), chickens (*n* = 2), a pig (*n* = 1), and a dog (*n* = 1). The *in silico* MLST and *E. coli* phylogenetic typing were performed using the MLST 2.0 (v2.0.4) program at the CGE website and the Clermont typing program (v21.03) provided by the website http://clermontyping.iame-research.center/ (Beghain et al., 2018). The assembly accession numbers of strains used in this study are summarized in Supplementary File 1.

#### In-depth Characterization of Intestinal Pathogenic MCR1-EC Strains Based on WGS

We conducted WGS for all intestinal pathogenic MCR1-EC strains isolated in this study. Total genomic DNA was extracted using the Nucleospin Microbial DNA kit (Macherey-Nagel, North Rhine-Westphalia, Germany) following the manufacturer’s instructions. Genomic DNA was sequenced *via* NextSeq® 500 technology (Illumina, California, United States). The nucleotide sequences have been submitted to the NCBI sequence read archive with the assigned Bioproject no. PRJNA757225. The sequence reads were assembled into contigs using the CLC Genomics Workbench program (Qiagen, Hilden, Germany) with default setting. The assembled contigs were analyzed using the bioinformatics tools of the Center for Genomic Epidemiology^2^ for the presence of resistance genes (ResFinder V4.1.), virulence factors (VirulenceFinder v2.0.), and plasmid replicon types (PlasmidFinder 2.1).

#### Genetic Relatedness Analysis Based on WGS

For genetic relatedness analysis based on WGS, we conducted core genome multi-locus sequence typing (cgMLST) to focus on the genetic relatedness between the core genomes of strains, not the genetic difference that occurs through the acquisition or loss of accessory genomes such as plasmids. The cgMLST was performed using the Ridom SeqSphere+ program (v8.2.0; Junemann et al., 2013). In this analysis, first, we conducted cgMLST among all 12 intestinal pathogenic MCR1-EC strains isolated from this study and 17 MCR1-EC strains isolated in South Korea published in the NCBI database to assess the genetic relatedness among strains isolated in South Korea. Second, for genetic relatedness analysis of global MCR1-EC strains, we performed cgMLST on MCR1-EC isolated from humans, pigs, and chickens worldwide and harboring a major clone type. Based on clonal distribution analysis, 154 strains carrying the major clone type ST10-A were identified among 1,652 MCR1-EC strains published in the NCBI database. Moreover, 80 strains were selected among 154 MCR1-EC isolates of clone type ST10-A using a simple random sampling procedure with Statistical Package for the Social Sciences (SPSS) program (v27.0, IBM SPSS Statistics for Windows, New York, United States). Then, the genetic relationships among 82 MCR1-EC strains harboring ST10-A (two intestinal pathogenic MCR1-EC strains isolated in this study and 80 MCR1-EC strains published in the NCBI database) were analyzed based on cgMLST. Then, we clustered strains with a genetic relatedness distance of less than 0.01 in cgMLST, and a total of eight clusters were identified.

### Statistical Analysis

All statistical analyses included in this study were conducted using the SPSS program (IBM SPSS Statistics for Windows). For analysis of MCR1-EC prevalence, we performed the weighted prevalence analysis of MCR1-EC [complex samples crosstabs (CSC) and complex samples logistic regression model (CSLRM)] based on the unbiased Horvitz-Thompson estimator (Horvitz and Thompson, 1952), setting farm as a cluster parameter since sampling probabilities for each swine farm were not equal. Weighted prevalence of MCR1-EC by stage and 95% confidence interval (95% CI) were calculated using CSC. In addition, differences in the prevalence of MCR1-EC according to swine stage were evaluated using the CSLRM setting stage as a covariate parameter.

For comparative analyses of antimicrobial resistance and virulence factors of MCR1-EC isolates by swine stages, the generalized estimating equation (GEE) was used for the calculation of odds ratios (ORs) and 95% CIs setting weaning stages as a reference. To adjust the farm-induced factors, farm was set as the “subject variable” and number of MCR1-EC strains per each farm was set as “within subject variables.” If the zero value of the cross-tab caused a problem in the GEE-based OR calculation, Fisher’s exact test was performed by adding 0.5 to each cell instead of GEE (Pagano and Gauvreau, 2018). To evaluate the correlation between antimicrobial resistance genes and the expected phenotypic resistance, Spearman’s correlation test (SCT) was performed.

## Results

### Prevalence of MCR1-EC Isolates According to Four Swine Production Stages

*Escherichia coli* strains carrying *mcr-1* strains were isolated from 55 of 331 pigs (16.6%), from four of 10 swine farms (Figure 2; Supplementary Table 1). The weighted prevalence of MCR1-EC was 11.6% (95% CI: 8.9%–15.0%), and weaning piglets had the highest weighted prevalence of MCR1-EC (17.9, 95% CI: 9.9%–30.3%). The second highest weighted prevalence MCR1-EC was identified in growing pigs (14.7, 95% CI: 9.2%–22.7%), followed by sows (8.3, 95% CI: 3.5%–18.4%), and finishing pigs (6.7, 95% CI: 3.4%–12.7%). There were no significant differences in the prevalence of MCR1-EC based on the four swine stages (CSLSM, *p* > 0.05).

We included 53 MCR1-EC stains for further analysis, since two MCR1-EC isolates were not recovered. All 53 MCR1-EC strains were found to carry *mcr-1.1* among 32 *mcr-1* variants (*mcr-1.1*–*mcr-1.32*), and *mcr-1.1* was encoded on either IncI2 (94.3%, 50/53) or IncX4 (5.7%, 3/53). MCR1-EC carrying both *mcr-1.1*-carrying IncI2 and IncX4 was not identified. In the conjugation assay of MCR1-EC strains, *mcr-1.1* was transferred from 90.6% (48/53) of donor strains to the recipient strain J53-Azi^R^.

**Figure 3:**
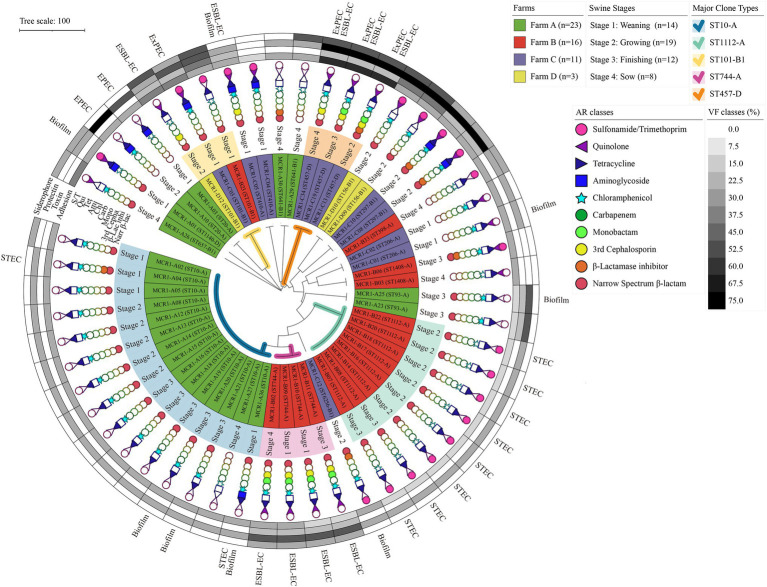
Antimicrobial resistance, genotypic/phenotypic virulence factors, and clone types of 53 MCR1-EC isolates from swine farms. Visualization was conducted using the online visualization tool iTOL (v6, https://itol.embl.de/). MCR1-EC, *Escherichia coli* carrying the mobilized colistin resistance gene *mcr-1.1*; EPEC, enteropathogenic *E. coli*; STEC, shiga toxin-producing *E. coli*; and ESBL-EC, extended-spectrum β-lactamase producing *E. coli*, Biofilm, *E. coli* with moderate/strong biofilm formation ability.

Among 53 MCR1-EC isolates, 16 strains (30.2%, 16/53) were identified as pathogenic *E. coli*, including InPEC (22.6%, 12/53) or ExPEC (7.5%, 4/53; Figure 3). Among 12 InPEC strains, 10 MCR1-EC (18.9%, 10/53) was identified as STEC and two strains (3.8%, 2/53) were identified as EPEC. Ten STEC were isolated from two weaning piglets, six growing pigs, and two finishing pigs. Two EPEC were isolated from one weaning piglet and one growing pig. Four ExPEC were isolated from one weaning piglet, one growing pig, one finishing pig, and one sow.

### Antimicrobial Resistance of MCR1-EC Isolates From Swine Farms

**Figure 4:**
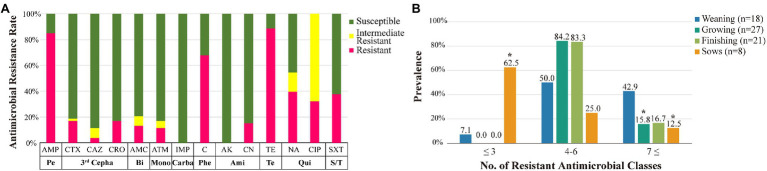
Antimicrobial susceptibility of MCR1-EC strains and prevalence of resistant MCR1-EC. Antimicrobial susceptibility of MCR1-EC from swine farms **(A)** and prevalence of MCR1-EC isolates resistant to different numbers of antimicrobial classes by swine production stage **(B)**. MCR1-EC, *Escherichia coli* carrying the mobilized colistin resistance gene *mcr-1.1*; Pe, broad spectrum penicillin class; third Cepha, third-generation cephalosporin class; Bi, β-lactamase inhibitor class; Mono, monobactam class; Carba, carbapenem class; Phe, phenicol class; Ami, aminoglycoside class; Te, tetracycline class; Qui, quinolone class; S/T, sulfonamide/trimethoprim class; AMP, ampicillin; CTX, cefotaxime; CAZ, ceftazidime; CRO, ceftriaxone; AMC, amoxicillin/clavulanate; ATM, aztreonam; IMP, imipenem; C, chloramphenicol; AK, amikacin; CN, gentamycin; TE, tetracycline; NA, nalidixic acid; CIP, ciprofloxacin; and SXT, sulfamethoxazole/trimethoprim. ^*^p < 0.05, significantly different prevalence relative to that of weaning piglets, calculated *via* GEEs.

All 53 MCR1-EC isolates were resistant to colistin, with MICs of 4 μg/ml (17.0%, 9/53) or 8 μg/ml (83.0%, 44/53). Through KBTs for 11 antimicrobial classes, 96.2% (51/53) of MCR1-EC strains exhibited MDR, showing resistance to three or more antimicrobial classes (average: 4.8 classes; Figure 3). Among the 14 antimicrobial agents tested, the resistance rate of tetracycline was highest (86.8%, 46/53), followed by that of ampicillin (81.1%, 43/53) and chloramphenicol (66.0%, 35/53; Figure 4A). Nine MCR1-EC strains (17.0%, 9/53) were resistant to cefotaxime and had a typical phenotype of ESBL. Imipenem- or amikacin-resistant MCR1-EC isolates were not found. In comparison by pathogenic *E. coli* types, ExPEC strains showed resistance to average 7.0 antimicrobial classes, and InPEC strains showed resistance to average 4.0 antimicrobial classes. The resistant rate of ExPEC strains against third generation cephalosporins was 75.0% (3/4), whereas, all InPEC strains were susceptible to third cephalosporins. The antimicrobial susceptibility results of InPEC, ExPEC, and commensal *E. coli* were described in Supplementary Table 4.

In the comparative analysis based on the four swine stages, the prevalence of isolates showing resistance to seven or more antimicrobial classes was highest in the weaning stage (42.9%, 6/14) compared to that in other stages, which was statistically significant compared to that in finishing pigs (OR: 3.8, 95% CI: 1.73–8.11, *p* < 0.05, GEE) and sows (OR: 5.25, 95% CI: 2.04–13.50, *p* < 0.05, GEE; Figure 4B). Meanwhile, the prevalence of isolates showing resistance to three or fewer antimicrobial classes was highest in pregnant sows (62.5%, 5/8), and it was significantly higher than that in weaning piglets (OR: 21.7, 95% CI: 8.77–53.50, *p* < 0.05, GEE). Compared to that in weaning pigs, the resistance rate of aminoglycoside was significantly lower in growing pigs (OR: 0.3, 95% CI: 0.12–0.51, *p* < 0.05, GEE), and the resistance rate of quinolone was significantly lower in finishing pigs (OR: 0.1, 95% CI: 0.07–0.18, *p* < 0.05, GEE; Table 1). Compared to those in weaning pigs, the resistance rate of chloramphenicol (OR: 0.1, 95% CI: 0.04–0.28, *p* < 0.05, GEE) and tetracycline (OR: 0.03, 95% CI: 0.001–0.78, *p* < 0.05, Fisher’s exact test) were significantly lower in pregnant sows.

**Table 1:** Antimicrobial resistance rate of *Escherichia coli* strains carrying *mcr-*1 (MCR1-EC) according to the pig productions stages.

Anti-microbial classes	Weaning piglets (reference)			Growing pigs			Finishing pigs			Pregnant sows		
	Rate (%)	OR (95% CI)	*p* value	Rate (%)	OR (95% CI)	*p* value	Rate (%)	OR (95% CI)	*p* value	Rate (%)	OR (95% CI)	*p* value
Pe	78.6	–	–	94.7	4.9 (0.62–39.16)	0.13	100.0	7.6^a^ (0.35–163.83)	0.19	50.0	0.3 (0.05–1.41)	0.12
Third Cepha	28.6	–	–	10.5	0.3 (0.02–3.64)	0.34	8.3	0.2 (0.01–5.24)	0.36	25.0	0.8 (0.15–4.64)	0.84
Carba	0.0	–	–	0.0	0.7^a^ (0.01–39.73)	0.88	0.0	1.2^a^ (0.02–62.85)	0.94	0.0	1.7^a^ (0.03–94.11)	0.79
Mono	21.4	–	–	5.3	0.2 (0.01–8.01)	0.40	8.3	0.3 (0.01–16.66)	0.58	12.5	0.5 (0.23–1.20)	0.13
Bi	7.1	–	–	21.1	3.5 (0.12–104.32)	0.47	8.3	1.2 (0.05–27.38)	0.92	12.5	1.9 (1.00–3.45)	0.05
Phe	85.7	–	–	63.2	0.3 (0.01–6.71)	0.44	75.0	0.5 (0.01–19.33)	0.71	37.5	0.1 (0.04–0.28)	<0.01^*^
Ami	42.9	–	–	15.8	0.3 (0.12–0.51)	<0.01^*^	0.0	0.5^a^ (0.002–1.06)	0.05	0.0	0.1^a^ (0.003–1.59)	0.10
Te	100.0	–	–	89.5	0.2^a^ (0.01–5.44)	0.37	100.0	0.9^a^ (0.02–46.71)	0.94	50.0	0.03^a^ (0.001–0.78)	0.03^†^
Qui	64.3	–	–	31.6	0.3 (0.03–1.97)	0.19	16.7	0.1 (0.07–0.18)	<0.01^*^	50.0	0.6 (0.21–1.49)	0.24
S/T	28.6	–	–	57.9	3.4 (0.43–27.61)	0.25	33.3	1.3 (0.06–28.35)	0.89	12.5	0.4 (0.03–4.30)	0.42

The odds ratio (OR), including 95% of confidential interval (95% CI) and *p* value, was calculated by generalized estimating equations (GEE).

a Where zeros cause problems in calculating OR or 95% CI, Fisher’s exact test was used in the calculations instead of GEE.

* *p* < 0.05, statistically significant based on GEE.

† *p* < 0.05, statistically significant based on Fisher’s exact test.

Pe, broad spectrum penicillin class; third Cepha, third cephalosporin class; Carba, carbapenem class; Mono, monobactam class; Bi, β-lactamase inhibitor class; Phe, phenicol class; Ami, aminoglycoside class; Te, tetracycline class; Qui, quinolone class; S/T, sulfonamide/trimethoprim; and class Poly, polymyxin class.

*Escherichia coli* strains carrying *mcr-1* carried a variety of antimicrobial resistance genes, including *tetA* (79.2%, 42/53, against tetracyclines), *floR* (69.8%, 37/53, against phenicols), *bla_TEM-family_* (58.8%, 31/53, against narrow-spectrum β-lactams), *sul2* (50.9%, 27/53, against sulfonamides), *qnrS1* (41.5%, 22/53, against quinolones), and *bla_CTX-M-55_* (17.1%, 9/53, against third Cephalosporins; Supplementary Table 5). Resistance genes were strongly associated with expected phenotypic resistance to all antimicrobial classes included in this study (*p* < 0.05, SCT), with the exception of quinolones. Among the 14 replicon types investigated in this study in 53 MCR1-EC isolates, the predominant replicon types were IncI2 (94.3%, 50/53), IncFIB (84.9%, 45/53), IncFII (67.9%, 36/53), and IncFIC (43.4%, 23/53; Supplementary Table 6).

### Genotypic and Phenotypic Virulence of MCR1-EC Isolates From Swine Farms

Among the four investigated virulence factor classes, all 53 MCR1-EC strains carried one or more adhesion-associated virulence factors, including *fimH* (90.6%, 48/53) and *csgA* (84.9%, 45/53) (Table 2). Toxin virulence factors were identified in 54.7% of MCR1-EC (29/53), with *hlyF* (26.4%, 14/53) and *astA* (7.5%, 4/53) present. Protectin virulence factors were identified in 90.6% of MCR1-EC (48/53), with *traT* (88.7%, 47/53), *ompT* (26.4%, 14/53), and *iss* (13.2%, 7/53) present. Siderophore virulence factors were identified in 35.8% of MCR1-EC (19/53), with *iutA* (26.4%, 14/53) and *iroNE.coli* (9.4%, 5/53) present. In addition, four (7.5%, 4/53) MCR1-EC were identified as having two UPEC-associated virulence factors, although this did not satisfy the criteria of UPEC (≥3 UPEC virulence factors). In the comparison based on the four swine stages, no significant differences were identified in the prevalence of the four virulence factor classes between stages (*p* > 0.05, GEE; Supplementary Table 7). In the biofilm assay, eight MCR1-EC strains (15.1%, 8/53) showed medium-to-strong biofilm formation (Figure 3), including four strains with strong biofilm formation and four strains with moderate biofilm formation. In contrast, 84.9% (45/53) of MCR1-EC showed weak biofilm formation.

**Table 2:** Analysis of pathogenic *E. coli*-associated virulence factors in MCR1-EC from swine farms.

Virulence factor classes	Virulence factors	Prevalence (%)	No. of positive MCR1-EC/No. of total MCR1-EC
Intestinal pathogenic *E. coli* (InPEC)	*stx2*	18.9	10/53
	*eaeA*	3.8	2/53
	*stx2* or *eaeA*	22.6	12/53
Extra-intestinal pathogenic *E. coli* (ExPEC)	*kpsMTII**^a^*	9.4	5/53
	*papC**^a^*	7.5	4/53
	*papAH**^a^*	7.5	4/53
	*sfa/focDE**^a^*	1.9	1/53
	*afa/draBC**^a^*	0.0	0/53
	Two and more ExPEC VFs	7.5	4/53
Uropathogenic *E. coli* (UPEC)	*fyuA**^b^*	7.5	4/53
	*chuA**^b^*	7.5	4/53
	*yfcV**^b^*	5.7	3/53
	*vat**^b^*	3.8	2/53
	Two and more UPEC VFs	7.5	4/53
Adhesion	*fimH*	90.6	48/53
	*csgA*	84.9	45/53
	*papC**^a^*	7.5	4/53
	*papAH**^a^*	7.5	4/53
	*yfcV**^b^*	5.7	3/53
	*sfa/focDE**^a^*	1.9	1/53
	*afa/draBC**^a^*	0.0	0/53
	*iha*	0.0	0/53
	Total adhesion (at least one)	100.0	53/53
Toxin	*hlyF*	26.4	14/53
	*astA*	7.5	4/53
	*vat**^b^*	3.8	2/53
	*pic*	0.0	0/53
	*aat*	0.0	0/53
	Total toxin (at least one)	32.1	17/53
Protectin	*traT*	88.7	47/53
	*ompT*	26.4	14/53
	*iss*	13.2	7/53
	*kpsMTII**^a^*	9.4	5/53
	Total protectin (at least one)	90.6	48/53
Siderophore	*iutA**^a^*	26.4	14/53
	*iroNE.coli*	9.4	5/53
	*fyuA**^b^*	7.5	4/53
	*chuA**^b^*	7.5	4/53
	Total siderophore (at least one)	35.8	19/53

a Virulence factors used for criteria of ExPEC; if positive for ≥2 of five key markers, including *papA* and/or *papC*, *sfa/focDE*, *afa/draBC*, *iutA*, and *kpsMTII*.

b Virulence factors used for criteria of UPEC; if positive for ≥3 of four key markers, including *vat*, *fyuA*, *chuA*, and *yfcV*.

### WGS-Based In-depth Characterization of Intestinal Pathogenic MCR1-EC Strains

**Figure 5:**
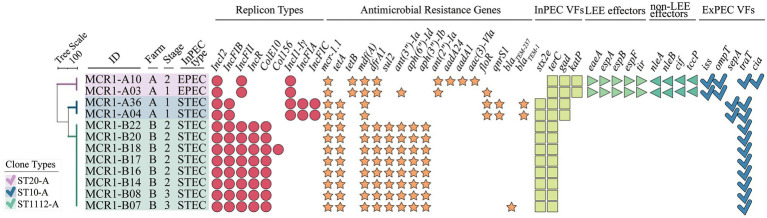
Whole-genome sequence (WGS)-based in-depth genetic characterization of 12 intestinal pathogenic MCR1-EC strains. The phylogenetic tree was constructed based on allele profiles of multi-locus sequence types (MLSTs) of 12 intestinal pathogenic MCR1-EC strains *via* the Unweighted Pair Group Method with Arithmetic means (UPGMA) method, calculating dice coefficients in the Bionumerics program (v6.6). Visualization was conducted using the online visualization tool iTOL (v6, https://itol.embl.de/). MCR1-EC, *Escherichia coli* carrying the mobilized colistin resistance gene *mcr-1.1*; Stage 1, weaning piglets; Stage 2, growing pigs; Stage 3, finishing pigs; Stage 4, pregnant sows; InPEC type, intestinal pathogenic *E. coli* type; EPEC, enteropathogenic *E. coli*; STEC, shiga toxin-producing *E. coli*; InPEC VFs, intestinal pathogenic *E. coli*-associated virulence factors; LEE effectors, locus of enterocyte effacement (LEE)-encoded effectors; non-LEE effectors, non-LEE-encoded effectors; and ExPEC VFs, extra-intestinal pathogenic *E. coli*-associated virulence factors.

All 10 STEC isolates harbored *stx2e*, and two EPEC strains harbored the locus of enterocyte effacement (LEE), including *eae*, *tir*, *esp*, and *nle* (Figure 5). Intestinal pathogenic MCR1-EC carried a variety of InPEC-associated virulence factors, including *terC* (100.0%, 12/12), *gad* (33.3%, 4/12), and *katP* (16.7%, 2/12). In addition, ExPEC-associated virulence factors, including *traT* (91.7%, 11/12), *ompT* (16.7%, 2/12), *iss*, (16.7%, 2/12), *sepA* (167%, 2/12), and *cia* (8.3%, 1/12), were also identified.

In the analysis of antimicrobial resistance genes, all 12 intestinal pathogenic MCR1-EC strains carried resistance genes to five or more antimicrobial classes, including *tetA/B* (100%, 12/12, against tetracyclines), *mdf(A)* (100%, 12/12, against macrolides), *dfrA1* (83.3%, 10/12, trimethoprims), *ant(3”)-Ia* (75.0%, 9/12, aminoglycosides), *sul2* (66.7%, 8/12, sulfonamides), *floR* (25.0%, 3/12, phenicols), and *bla_TEM-family_* (25.0%, 3/12, narrow-spectrum β-lactams). All carried IncI2, accompanying by a variety of replicon types, including IncFIB (83.3%, 10/12), IncFII (83.3%, 10/12), IncR (66.7%, 8/12), ColE10 (66.7%, 8/12), and IncI1-Iγ (33.3%, 4/12).

In the comparative genomic analysis based on swine production stages, intestinal pathogenic MCR1-EC showed highly shared virulence factor characteristics between strains with the same clone type. In addition, the patterns of replicon types and antimicrobial resistance genes were also identical with slight differences between strains with the same clone types.

### Genetic Relatedness Analysis of MCR1-EC Strains Based on Clone Types and WGS

#### Clonal Distribution Analysis of MCR1-EC From This Study and the NCBI Database

**Figure 6:**
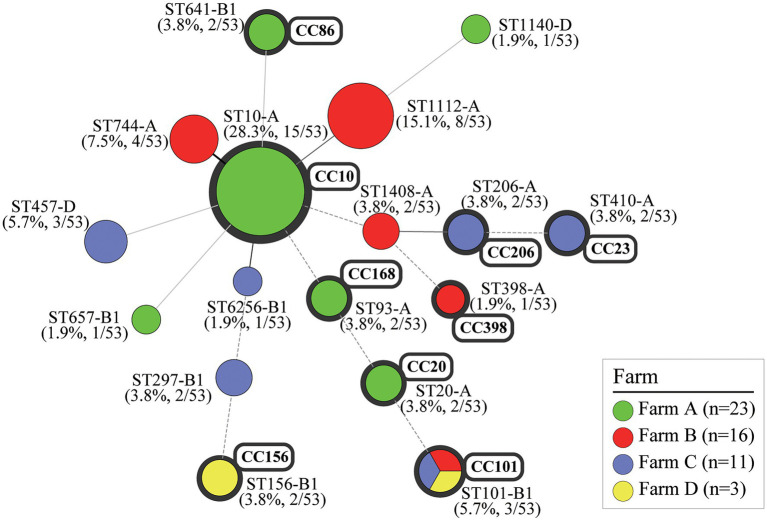
Clonal distribution of MCR1-EC isolates: Minimum spanning tree (MST) based on MLST allele profiles. The MST was constructed using the Bionumerics program (v6.6). The colors of nodes correspond to the four swine farms. The upper number shows the sequence type of each node, and the lower number in parentheses indicates percentages for each node. The size of the node indicates the number of strains belonging to the sequence type (ST)-phylogroup (PG) type. The gray shaded area represents the clonal complex (CC). The branch line types represent differences in the number of alleles as follows: bold solid line (one allele), thin solid line (2–3 alleles), dashed line (four alleles), and dotted line (above five alleles). MCR1-EC, *Escherichia coli* carrying the mobilized colistin resistance gene *mcr-1.1*; MLST, multi-locus sequence typing.

Among 53 MCR1-EC strains, 38 strains were identified as *E. coli* phylogenetic group A (71.7%, 38/53), 11 strains (20.8%) were identified as group B1, and four strains (7.5%) were identified as group D (Figure 6). In total, 17 clone types were identified among 53 MCR1-EC strains isolated in this study, and the major clone types were ST10-A (28.3%, 15/53), ST1112-A (15.1%, 8/53), ST744-A (7.5%, 4/53), ST101-B1 (5.7%, 3/53), and ST457-D (5.7%, 3/53). The other clone types included only one or two MCR1-EC strains. In a comparison by swine farm, all clone types were not shared between pig farms with the exception of ST101-B1, which was isolated from three pig farms. In a comparison by swine production stage, the clone types were shared between pigs of different stages within farms (Figure 3).

In the clonal distribution analysis of MCR1-EC published in the NCBI database, 17 MCR1-EC strains derived from South Korea harbored 15 clone types, including ST10-A (11.8%, 2/17) and ST11124-A (11.8%, 2/17; Supplementary File 1). In the clonal distribution analysis of human-, pig-, and chicken-derived 1,652 MCR1-EC, 248 clone types were identified among 940 human-derived MCR1-EC, and major clone types were ST10-A (9.6%, 90/940), ST152-A (3.5%, 33/940), ST206-A (3.0%, 28/940), and ST101-B1 (2.9%, 27/940). Among 266 pig-derived MCR1-EC strains, 101 clone types were identified, and major types were ST10-A (11.3%, 30/268), ST206-A (4.1%, 11/266), and ST101-B1 (3.8%, 10/266). Among 446 chicken-derived MCR1-EC isolates, 118 clone types were identified and major types were ST10-A (7.6%, 34/446), ST156-B1 (6.7%, 30/446), and ST93-A (4.7%, 21/446).

#### Genetic Relatedness Analysis Based on cgMLST Between MCR1-EC Strains From This Study and the NCBI Database

**Figure 7:**
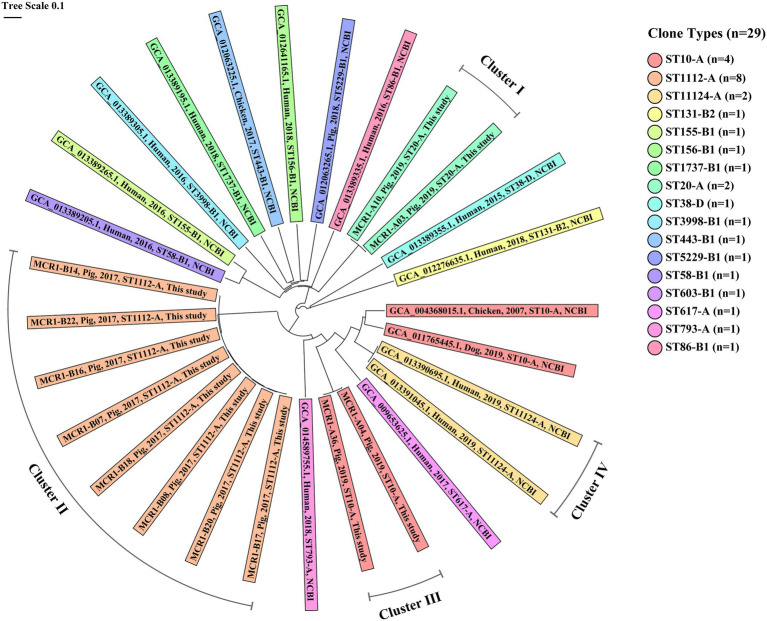
Core genome MLST (cgMLST)-based genetic relatedness between 29 MCR1-EC isolates from South Korea. Data comprise 12 intestinal pathogenic MCR1-EC strains isolated in this study and 17 MCR1-EC strains isolated from South Korea, published in the National Center for Biotechnology Information (NCBI) GenBank database. The phylogenetic tree based on cgMLST was constructed using the neighbor-joining algorithm with default parameters implemented in the Ridom SeqSphere+ program (v8.2.0). The color of shades corresponds to each clone type. The contents of shades include, from left to right, the assembly accession number, host, isolation date, clone type, and data source (this study or NCBI database) for each strain. Four clusters consist of MCR1-EC strains with a relatedness distance value less than 0.01. MCR1-EC, *Escherichia coli* carrying the mobilized colistin resistance gene *mcr-1.1*; MLST, multi-locus sequence typing.

In the cgMLST-based genetic relatedness analysis of intestinal pathogenic MCR1-EC isolated from this study and South Korea-derived MCR1-EC published on the NCBI database, the genetic relatedness distances between strains ranged from 0.000 to 0.961 (average 0.720, 95% CI: 0.694–0.746; Figure 7). We clustered strains with a genetic relatedness distance of less than 0.01 in cgMLST, and a total of four clusters (clusters I–IV) were identified. Cluster I included two ST20-A MCR1-EC strains (MCR1-A03 and MCR1-A10) isolated from one weaning piglet and one growing pig in Farm A. Cluster II included eight ST1112-A MCR1-EC strains (MCR1-B07, B08, B14, B16, B17, B18, B20, and B22) isolated from six growing and two finishing pigs in Farm B. Cluster III included two ST10-A MCR1-EC strains (MCR1-A04 and MCR1-A36) isolated from two weaning piglets in Farm A. Cluster IV included two ST11124-A MCR1-EC strains (GCA_013390695.1 and GCA_013391045.1) published in the NCBI database. All strains of four clusters were identified as being isolated from individuals from the same farm or hospital. According to the metadata in the original report, two South Korean-derived ST11124-A MCR1-EC strains in cluster IV were reported to be isolated from two patients in the same hospital but at different collection times for each strain (Kim et al., 2021). Except for MCR1-EC strains belonging to four clusters, the genetic relatedness distance was confirmed to have an average value of 0.771 (95% CI: 0.752–0.790), and the average value was 0.397 (95% CI: 0.317–0.477) even among six MCR1-EC isolates carrying the same clone type, ST10-A.

**Figure 8:**
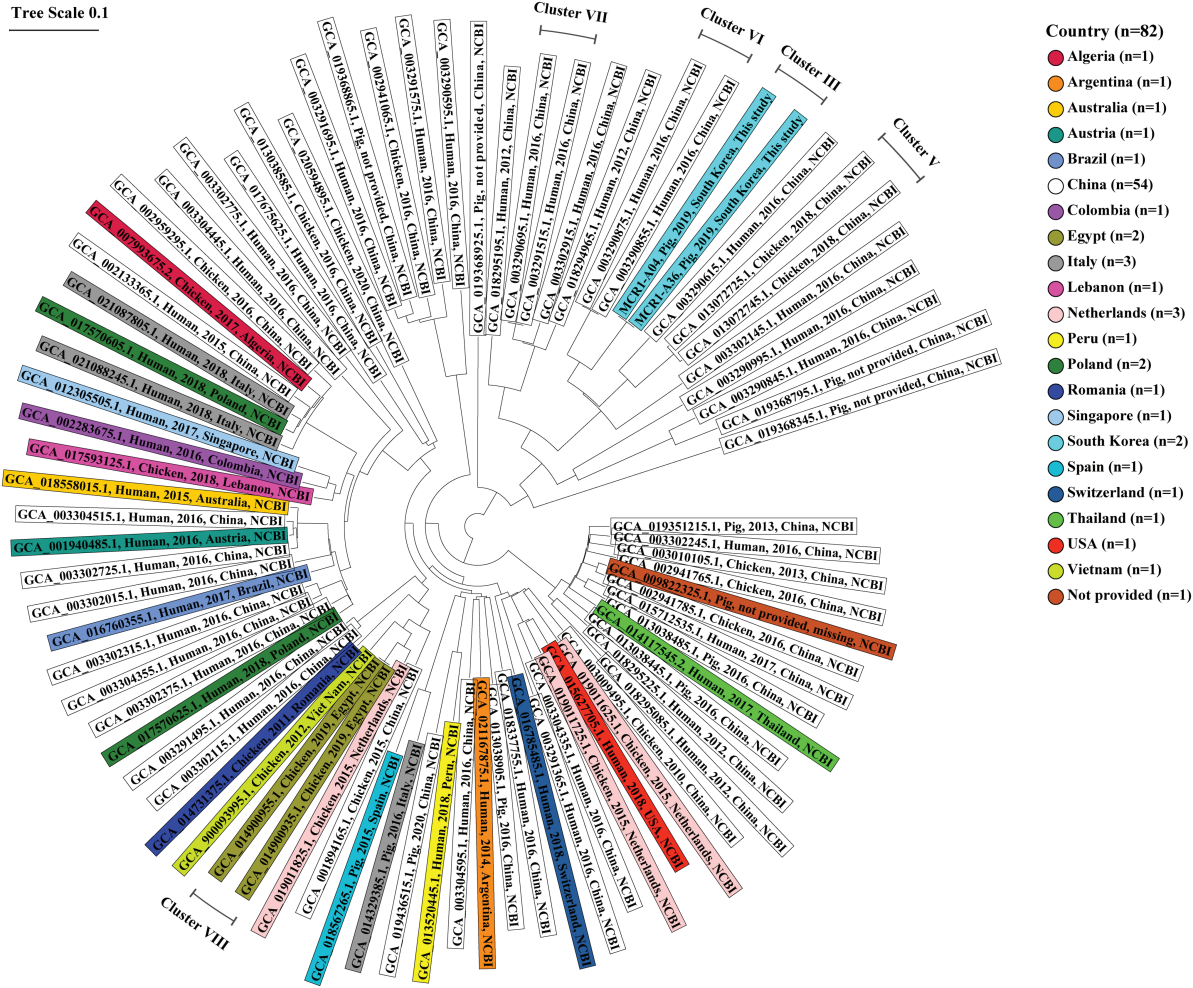
Core genome multi-locus sequence typing-based genetic relatedness of 82 ST10-A MCR1-EC strains isolated from humans, pigs, and chickens worldwide. Strains were derived from the NCBI GenBank database. The phylogenetic tree based on cgMLST was constructed using the neighbor-joining algorithm with default parameters implemented in the Ridom SeqSphere+ program (v8.2.0). The shaded color corresponds to the country where the strain was isolated. The contents of shades include, from left to right, the assembly accession number, host, isolation date, isolation country, and data source (this study or NCBI database) for each strain. Five clusters consist of MCR1-EC isolates with a relatedness distance value of less than 0.01. MCR1-EC, *Escherichia coli* carrying the mobilized colistin resistance gene *mcr-1.1*; cgMLST, core genome multi-locus sequence typing.

In the genetic relatedness analysis of 82 ST10-A MCR1-EC strains from humans, chickens, and pigs worldwide, the genetic relatedness distance between strains ranged from 0.000 to 0.525 (average 0.309, 95% CI: 0.305–0.312; Figure 8). We clustered strains with a genetic relatedness distance of less than 0.01 in cgMLST, and a total of five clusters (cluster III, V, VI, VII, and VIII) were identified. Cluster III included two MCR1-EC strains (MCR1-A04 and MCR1-A36) isolated from Farm A in this study. Cluster V included two chicken-derived strains (GCA_013072745.1 and GCA_013072725.1) from China. Cluster VI included two human-derived strains (GCA_003290855.1 and GCA_003290875.1) from China. Cluster VII included two human-derived strains (GCA_003291515.1 and GCA_003290695.1) from China. Cluster VIII included two chicken-derived strains (GCA_014900955.1 and GCA_014900935.1) from China. According to the metadata in the original report, MCR1-EC, belonging to the four clusters V, VI, VII, and VIII, was isolated from individuals in the same hospital or farm, with strains in the same cluster (Shen et al., 2018; Soliman et al., 2021). Except for MCR1-EC isolates belonging to four clusters, the genetic relatedness distance between the other MCR1-EC isolates was confirmed to have an average value of 0.309 (95% CI: 0.305–0.313).

## Discussion

The global emergence and spread of MCR1-EC represent a serious threat for public health (WHO, 2018). Although the use of colistin for the prevention of swine colibacillosis has been banned from multiple countries worldwide since 2016, colistin has been generally used for the treatment of swine diseases, leading to an increased prevalence of MCR1-EC in swine farms worldwide including South Korea (Malhotra-Kumar et al., 2016; Tong et al., 2018; Kyung-Hyo et al., 2020; Liu et al., 2020; Mechesso et al., 2020; Nakano et al., 2021). In this study, the weighted prevalence of MCR1-EC was 11.6% (95% CI: 8.9%–15.0%) and it was comparable with that in previous reports conducted from Belgium (13.2%; Malhotra-Kumar et al., 2016), Japan (20.4%; Nakano et al., 2021), Taiwan (29.2%; Liu et al., 2020), and China (76.2%; Tong et al., 2018). Comparison of the four swine production stages showed that weaning piglets exhibited the highest prevalence of MCR1-EC compared with pigs at other stages. This result was consistent with that of previous studies conducted worldwide, in which MCR1-EC was isolated mainly from weaning piglets (Malhotra-Kumar et al., 2016; Tong et al., 2018; Wang et al., 2018b; Kyung-Hyo et al., 2020; Liu et al., 2020; Mechesso et al., 2020; Nakano et al., 2021). Recent studies on the occurrence of MCR1-EC in pigs following the cessation of colistin use have proposed a positive correlation between colistin administration and prevalence of MCR1-EC in swine farms (Randall et al., 2018; Shen et al., 2020). Considering that colistin has been reported to be mainly prescribed in weaning stages for the treatment of swine colibacillosis, which exhibits higher incidence during the weaning stage (Callens et al., 2012; Sjolund et al., 2016; Korsgaard et al., 2020), high colistin at weaning stage could be one of the important causes for the high prevalence of MCR1-EC at this stage.

Notably, 96.2% of MCR1-EC exhibited MDR, with resistance against average 4.8 antimicrobial classes. Furthermore, all MCR1-EC showed intermediate-to-resistance to ciprofloxacin and 17.0% of strains showed an ESBL phenotype as well as carried *bla_CTX-M-55_*. The *bla_CTX-M-55_* gene has been globally reported as an ESBL genotype that confers resistance to third-generation cephalosporins, and it has been found in various hosts including humans and food-animals (Lv et al., 2013). In the comparative analysis based on the four swine stages, it was found that the weaning piglets exhibited significantly higher resistance rates to various antimicrobial agents than other stages, especially sow. This result suggests that the antimicrobial resistance found in weaning piglets may not be inherited from sow. According to studies on the use of antibiotics throughout the swine production cycle, over than 70% of the total antimicrobial agents used in the swine industry have been prescribed between birth and 10 weeks of age (Callens et al., 2012; Sjolund et al., 2016; Korsgaard et al., 2020). Antibiotic selective pressure has been reported to play significant role in the increasing prevalence of resistant bacteria (Randall et al., 2018). Wu et al. (2018a) suggested that colistin and β-lactam antibiotics have been commonly prescribed together in food-animal husbandry, and resistance to colistin and third generation cephalosporins emerged and increased together under the heavy selection pressure of antibiotics over the last few decades. In our previous study, we investigated the prevalence of ESBL-producing *E. coli* (ESBL-EC) in pig farms, which revealed that the prevalence of ESBL-EC was significantly higher in weaning piglets compared with that in other stages and sow showed the lowest prevalence of ESBL-EC (Lee et al., 2021). In addition, interestingly, the prevalence of ESBL-EC in the four farms, where MCR1-EC was found in the present study, was significantly higher (76.4%; OR: 3.8, 95% CI: 1.73–8.14, *p* < 0.05, GEE) than in farms where MCR1-EC was not identified (46.2%). Hence, these findings were consistent with the conclusion of Wu et al. (2018a). Our study suggests that weaning piglets could act as an important reservoir for MDR bacteria, including ESBL-EC and MCR1-EC. The potential of MDR bacterial transmission from food-animal husbandry to humans and vice versa has been continuously proposed by various studies (Liu et al., 2016; Hadjadj et al., 2017; Wu et al., 2018a). Considering that both third generation cephalosporins and colistin are classified as critically important antimicrobial agents for livestock and humans (WHO, 2018), the high prevalence of MDR bacteria carrying both *mcr-1.1* and *bla_CTX-M-55_* implies the potential for the emergence of MDR pathogens, which can hardly be treated, even by last resort antimicrobials. Collectively, we suggest that pig farms, which are important reservoirs of MDR bacteria, require special attention at the weaning stage to control crucial bacteria, such as MCR1-EC and ESBL-EC.

Swine colibacillosis is one of the major swine diseases impacting the global swine industry and is associated with huge economic losses; edema disease (ED) and post-weaning diarrhea (PWD) belong to the classification of swine colibacillosis (Luppi, 2017). Given that colistin has been considered a recommended treatment for swine colibacillosis and InPECs are major causative bacteria of swine colibacillosis (Luppi, 2017), the presence of intestinal pathogenic MCR1-EC in pig husbandry could represent a major challenge for the swine industry. In this study, 22.6% of MCR1-EC strains were identified as InPECs including STEC carrying *stx2e* and EPEC carrying LEE-encoded virulence factors. The *stx2e* gene is key virulence factor causing damage to arterioles and edema at various sites, eventually leading to death associated with ED (Casanova et al., 2018). The LEE-encoded VFs are responsible for the characteristic histopathological lesion of PWD, termed attaching/effacing lesions (Rhouma et al., 2017). Among various identified virulence factors from intestinal pathogenic MCR1-EC strains, the presence of *katP* might especially increase the risk presented by the strains, since it has been reported to promote the virulence of InPECs by supporting their colonization of the host intestine (Brunder et al., 1996). In addition to virulence factors, all intestinal pathogenic MCR1-EC strains were identified as MDR bacteria harboring five or more antimicrobial class resistance genes. Comparative genomic analysis according to the stage of pig development revealed that the genetic characteristics of the intestinal pathogenic MCR1-EC strains were highly shared among pigs at different stages, suggesting that there is a high potential for the transmission of intestinal pathogenic MCR1-EC within farms. Although *E. coli* is a major organism carrying *mcr* genes, other *Enterobacterales* species have also been reported to carry the *mcr* genes and inhabit the intestinal tract of pigs (Lima et al., 2019; Phetburom et al., 2021). In addition, *mcr* genes have been reported to be highly transferred from *E. coli* to other pathogens, causing swine diseases, such as *Salmonella*, *Klebsiella*, and *Pseudomonas* (Kim et al., 2019). Thus, in cases of swine diseases caused by pathogens harboring these multiple virulence factors and MDR, the prescription of colistin may simply impose selection pressure, leading to disease treatment failure and the spread of colistin resistance in swine farms. To the control these highly virulent and MDR pathogens, it may be necessary to establish a strategy based on in-depth characterization, such as WGS analysis, rather than blindly using antibiotics for the treatment of swine diseases.

In the investigation of genotypic and phenotypic virulent characteristics, MCR1-EC isolates carried multiple ExPEC-associated virulence factors, including *traT*, *hlyF*, and *kpsMTII*, and four MCR1-EC isolates were identified as ExPEC. A high rate of ExPEC-associated virulence factors has been reported to correspond with high potential for survival in the harsh environments and pathogenicity of the bacteria against the host immune system (Pitout, 2012). The expression of TraT protein, an outer membrane lipoprotein, has been linked to improved serum resistance (Nilius and Savage, 1984). The hemolysin production regulator *hlyF* create pores in the membrane of host cells, which increasing the permeability of host cells and ending cell lysis (Bhakdi et al., 1988). The *kpsMTII* has been reported to encoding capsular polysaccharides acting protect the bacteria from environment by covering bacteria and helping to form biofilm (Antao et al., 2009). In addition to genotypic virulence, eight MCR1-EC strains showed moderate-to-strong biofilm formation capacity. Biofilm formation has been reported to confer a fitness advantage to bacteria by enhancing their survivability, increasing their virulence, and facilitating their ability to acquire virulence and antibiotic resistance genes during horizontal gene transmission owing to their high microbial density (Donlan and Costerton, 2002; Schroeder et al., 2017) Based on fitness advantages, such as strong biofilm formation or harboring multiple ExPEC virulence factors, MCR1-EC could survive better in an environment of swine farm husbandry and continuously exist through a repeated cycle, which involves the shedding from swine through feces, survival in the farm environment, and reintroduction to swine. In addition, although MCR1-EC might not be directly transmitted from pig farms to humans through the food-chain, these fitness advantages could provide MCR1-EC strains possibility to survive better in the food-chain and serve as an important source of *mcr-1.1* for various other bacteria in food-chains through genetic transmission mechanisms, such as conjugation.

In the analysis of clonal distribution of MCR1-EC, ST10-A was the most prevalent clone type of MCR1-EC strains in this study, as well as in the human, pig, and chicken-derived MCR1-EC strains described on the NCBI database. However, ST10-A represented only 28.3% of the MCR1-EC samples isolated in this study and 9.8% of 1,562 MCR1-EC samples described in the NCBI database. Other clone types, such as ST101-B1, ST744-A, and ST206-A, also accounted for a significant proportion of total strains. Consistently, the epidemiological analyses of MCR1-EC global clonal distribution revealed that ST10-A was the most prevalent clone type of MCR1-EC in humans and food-animals, whereas the other clone types also accounted for a significant proportion among total strains (Malhotra-Kumar et al., 2016; Matamoros et al., 2017; Garcia et al., 2018; Tong et al., 2018; Liu et al., 2020; Nakano et al., 2021). Furthermore, one recent study in Thailand showed that the dominant clone type of MCR1-EC in swine farms was ST101, followed by ST10 (Khanawapee et al., 2021). Interestingly, the results of clonal distribution analysis of MCR1-EC isolated from pig farms in this study revealed that the clone types were highly shared among MCR1-EC strains isolated from the same farm, but not between farms. Comparison by swine farms showed that all clone types, including the most predominant clone type ST10-A, were not shared between pig farms with the exception of ST101-B1, which was identified in three pig farms. Collectively, our study suggests that clonal types of MCR1-EC may vary widely between studies, and that it may be shared within closed environments such as a pig farm, but not between environments such as different pig farms or food-chains. Hence, this suggestion may imply that that clonal expansion alone may not have a direct role in MCR1-EC propagation between environments.

The cgMLST-based genetic relatedness analysis of intestinal pathogenic MCR1-EC strains isolated in this study, as well as those published in the NCBI database, revealed that MCR1-EC strains isolated from individuals within closed environment (such as hospitals or farms) were highly clustered, showing a genetic distance lower than 0.01. Noteworthy, clustered strains were isolated within the same hospitals or farms, but in separate spaces or at different time points. According to the original metadata of the two strains in cluster IV, they were isolated from patients in the same hospital but with a time interval of 2 months (Kim et al., 2021). In addition, two strains in cluster I and eight strains in cluster II, isolated in the present study, were isolated from different swine stages, which mean that they were isolated from pigs living in separate barns, including weaning, growing, and finishing barns. These results suggest that the clonal expansion may have a relatively high contribution to the propagation of MCR1-EC between individuals in closed environments. Since *mcr-1* is mainly transmitted by plasmids, the important role of genetic transferability of *mcr-1* in the spread of MCR1-EC has been continuously highlighted in various studies. However, genetic transfer essentially presupposes the transfer of strains and bacteria-to-bacteria interactions under favorable conditions, such as physical distance between strains, nutrition, and environmental conditions, among others (Virolle et al., 2020), which suggests that bacterial transmission also provides a crucial basis for the spread of MCR1-EC. It was previously reported that bacterial transmission between swine production stages within farms may probably occur through farm worker/veterinarian handling, equipment contamination, and transference of manure excretions between different stage barns (Fromm et al., 2014; Schmithausen et al., 2015). Our results suggest that bacterial cross-infection between different stages, pigs may act as an important risk factor for the prevalence of MCR1-EC. Swine farms have been continuously reported as an important reservoir of MCR1-EC (Malhotra-Kumar et al., 2016; Tong et al., 2018; Liu et al., 2020; Nakano et al., 2021). Our findings highlight that efforts to reduce bacterial cross-infection between stages are imperative to control MCR1-EC prevalence in swine farms, one of major reservoir of MCR1-EC.

Among reported *mcr* variants, the present study focused on the most predominant variant type, *mcr-1*. Recent studies have shown that the mobile genetic elements associated with the *mcr* genes may differ between variant types, which may lead to different genotypic and phenotypic traits in bacteria (Yin et al., 2017; Wang et al., 2018a,b,c; Lu et al., 2019; Yang et al., 2021). In this study, we conducted the comparative analysis of prevalence, characteristics, and clonal distribution of MCR1-EC according to swine production stages by excluding other *mcr* variants, which could be potential confounding factors. For further study, it would be interesting to analyze the characteristic differences of the other major *mcr* variants, such as *mcr-3* or *mcr-9*, according to food-animal production stages in livestock husbandry. Based on the 2017 demographic report of the Korean Statistical Information Service of Statistics, we analyzed the prevalence, characteristics, and clonal distribution of MCR1-EC according to four swine production stages in 10 swine farms, which were located in the provinces with the largest number of farms in South Korea. Overall, MCR1-EC was identified in four farms among the 10 swine farms investigated, of which three farms with MCR1-EC incidence were located in Gyeonggi-do. This result suggests that this study may not reflect the national prevalence of MCR1-EC, but regional characteristics. Further studies based on the national antimicrobial monitoring system by expanding the target farms and sampling size may help further describe the nationwide characteristics of MCR1-EC incidence and prevalence according to swine production stages.

## Conclusion

In conclusion, our study results showed that MCR1-EC isolates having MDR (e.g., against quinolones and ESBLs) were distributed throughout swine production stages in farms, with the highest prevalence at the weaning stage. Weaning stage-derived MCR1-EC showed a significantly higher resistance rate than those from other stages. MCR1-EC with pathogenic advantages (e.g., InPEC/ExPEC-associated virulence factors or robust biofilm formation) were identified from all pig stages and accounted for nearly half of the total strains. Genetic relatedness analysis based on MLST and cgMLST proposed a high potential for cross-infection of MCR1-EC within closed environment such as livestock farms as well as human hospitals. Our results highlight the need to establish MCR1-EC control plans in swine farms based on an in-depth understanding of MCR1-EC characteristics according to swine production stages, focusing especially on the weaning stages.

## Data Availability Statement

The data presented in the study are deposited in the NCBI sequence read archive repository, with bioproject accession no. PRJNA757225.

## Author Contributions

SL was a major contributor, both in experiments and writing the manuscript. SL and SC conceived and designed the study. SL, J-UA, HS, and SY performed the sampling and experiments. SL, J-UA, JW, J-HL, and SR analyzed the data. SL, W-HK, and SC prepared and reviewed the manuscript. All authors contributed to the article and approved the submitted version.

## Funding

This research was supported by the National Research Foundation of Korea (NRF-2021R1A2C2005907).

## Conflict of Interest

The authors declare that the research was conducted in the absence of any commercial or financial relationships that could be construed as a potential conflict of interest.

## Publisher’s Note

All claims expressed in this article are solely those of the authors and do not necessarily represent those of their affiliated organizations, or those of the publisher, the editors and the reviewers. Any product that may be evaluated in this article, or claim that may be made by its manufacturer, is not guaranteed or endorsed by the publisher.

## Footnotes

^1^ https://pubmlst.org/

^2^ http://www.genomicepidemiology.org/
