# Supplementary material for: Prevalence, Characteristics, and Clonal Distribution of Escherichia coli Carrying Mobilized Colistin Resistance Gene mcr-1.1 in Swine Farms and Their Differences According to Swine Production Stages
Source: Front Microbiol. 2022 May 6;13:873856. doi: 10.3389/fmicb.2022.873856 (PMC9121016; doi:10.3389/fmicb.2022.873856)
Supplement: Supplementary file 2 [file Data_Sheet_1.docx]

**Supplementary Table 1. Farm and sampling information for eleven swine farms included in this study and prevalence of *Escherichia coli* carrying *mcr-1* (MCR1-EC) across farms.**

| Farm Information | Farm ID | Total | Farm A | Farm B | Farm C | Farm D | Farm E | Farm F | Farm G | Farm H | Farm I | Farm J |
| --- | --- | --- | --- | --- | --- | --- | --- | --- | --- | --- | --- | --- |
|  | Province | - | Gyeonggi-  do | Gyeonggi-  do | Gyeongsang-nam-do | Gyeonggi-  do | Jeolla-nam-do | Jeolla-nam-do | Chungcheong-nam-do | Jeolla-buk-do | Chungcheong-nam-do | Chungcheong-nam-do |
|  | Sample collection time | - | Jul 2019 | May 2017 | May 2018 | Jan 2019 | Jul 2017 | Aug 2017 | Apr 2018 | May 2018 | Jul 2019 | Jul 2019 |
|  | Prevalence of ESBL-EC ^a^ | - | 64.7% | 82.4% | 58.8% | 73.1% | 61.8% | 23.5% | 17.6% | 51.5% | 36.7% | 50.0% |
| Prevalence  of MCR1-EC |  | No. of MCR1-EC-positive samples / No. of total samples  (unweighted prevalence) | | | | | | | | | | |
|  | Weaning  pigs | 14/59  (23.7%) | 6/6  (100.0%) | 3/6  (50.0%) | 5/6  (83.3%) | 0/5  (0.0%) | 0/6 | 0/6 | 0/6 | 0/6 | 0/6 | 0/6 |
|  | Growing  pigs | 20/108  (18.5%) | 6/11  (54.5%) | 6/10  (60.0%) | 4/11  (36.4%) | 4/10  (40.0%) | 0/11 | 0/11 | 0/11 | 0/11 | 0/11 | 0/11 |
|  | Finishing  pigs | 13/107  (12.1%) | 7/11  (63.6%) | 4/11  (36.4%) | 1/11  (9.1%) | 1/8  (12.5%) | 0/11 | 0/11 | 0/11 | 0/11 | 0/11 | 0/11 |
|  | Pregnant  sows | 8/57  (14.0%) | 4/6  (66.7%) | 3/6  (50.0%) | 1/6  (16.7%) | 0/3  (0.0%) | 0/6 | 0/6 | 0/6 | 0/6 | 0/6 | 0/6 |
|  | Total | 55/331  (16.6%) | 23/34  (67.6%) | 16/33  (48.5%) | 11/34  (32.4%) | 5/26  (19.2%) | 0/34 | 0/34 | 0/34 | 0/34 | 0/34 | 0/34 |

Abbreviation. No., Number; ESBL-EC, extended-spectrum β-lactamase-producing *Escherichia coli.*

^a^ Prevalence of ESBL-EC in swine farms was investigated in our previous study [1].

**Supplementary Table 2. Oligonucleotide sequences and annealing temperature of primers used for antimicrobial resistance genes and replicon typing**

| Function | Genes |  | Nucleotide sequence | Amplicon size (bp) | Annealing temp. (℃) | Reference |
| --- | --- | --- | --- | --- | --- | --- |
| Replicon Typing of *mcr-1-*carrying Plasmids | *mcr-1* | F | CCGTAATTATCCCACCGTTT | 1,719 | 60 | This study |
|  |  | R | CGCCCATAATACGAATGGAG |  |  |  |
|  | *mcr-1*-IncI2 | F | AACGGTGTCTATCTACATGGTAT | 1,330 | 60 | [2] |
|  |  | R | ACTTAGCGATCTCGTTGTT |  |  |  |
|  | *mcr-1*-IncX4 | F | AACGGTGTCTATCTACATGGTAT | 1,674 | 60 | [2] |
|  |  | R | CATTGAATTTGTTCGTCCTC |  |  |  |
| ESBL genotypes | *bla_CTX-M-1_* group | F | GTTACAATGTGTGAGAAGCAG | 1,041 | 60 | [3] |
|  |  | R | CCGTTTCCGCTATTACAAAC |  |  |  |
|  | *bla_CTX-M-2_* group | F | CGACGCTACCCCTGCTATT | 832 | 60 | [3] |
|  |  | R | CAGAAACCGTGGGTTACGAT |  |  |  |
|  | *bla_CTX-M-8_* group | F | GGCGCTGGAGAAAAGCAG | 862 | 60 | [3] |
|  |  | R | GGTTTTATCCCCGACAACC |  |  |  |
|  | *bla_CTX-M-9_* group | F | GTGACAAAGAGAGTGCAACGG | 857 | 60 | [3] |
|  |  | R | ATGATTCTCGCCGCTGAAGCC |  |  |  |
|  | *bla_CTX-M-25_* group | F | GCACGATGACATTCGGG | 327 | 60 | [3] |
|  |  | R | AACCCACGATGTGGGTAGC |  |  |  |
|  | *bla_CMY_* | F | AACACACTGATTGCGTCTGAC | 1,226 | 60 | [3] |
|  |  | R | CTGGGCCTCATCGTCAGTTA |  |  |  |
|  | *bla_SHV_* | F | TCGCCTGTGTATTATCTCCC | 768 | 54 | [3] |
|  |  | R | CGCAGATAAATCACCACAATG |  |  |  |
|  | *bla_TEM_* | F | TCCGCTCATGAGACAATAACC | 1,057 | 58 | [3] |
|  |  | R | ACGCTCAGTGGAACGAAAAC |  |  |  |
|  | *bla_OXA_* | F | ACACAATACATATCAACTTCGC | 813 | 60 | [3] |
|  |  | R | AGTGTGTTTAGAATGGTGATC |  |  |  |
| Phylogenetic group | *yjaA* | F | CAAACGTGAAGTGTCAGGAG | 288 | 55 | [4] |
|  |  | R | AATGCGTTCCTCAACCTGTG |  |  |  |
|  | *chuA* | F | ATGGTACCGGACGAACCAAC | 211 | 55 | [4] |
|  |  | R | TGCCGCCAGTACCAAAGACA |  |  |  |
|  | *tspE4.C2* | F | CACTATTCGTAAGGTCATCC | 152 | 55 | [4] |
|  |  | R | AGTTTATCGCTGCGGGTCGC |  |  |  |
|  | *AceK.f* | F | AACGCTATTCGCCAGCTTGC | 400 | 55 | [4] |
|  | *ArpA1.r* | R | TCTCCCCATACCGTACGCTA |  |  |  |
|  | *ArpAgpE.f* | F | GATTCCATCTTGTCAAAATATGCC | 301 | 55 | [4] |
|  | *ArpAgpE.r* | R | GAAAAGAAAAAGAATTCCCAAGAG |  |  |  |
|  | *trpAgpC.1* | F | AGTTTTATGCCCAGTGCGAG | 219 | 55 | [4] |
|  | *trpAgpC.2* | R | TCTGCGCCGGTCACGCCC |  |  |  |
|  | *trpBA.f* | F | CGGCGATAAAGACATCTTCAC | 489 | 55 | [4] |
|  | *trpBA.r* | R | GCAACGCGGCCTGGCGGAAG |  |  |  |
| Antimicrobial resistance | *catA* | F | AGTTGCTCAATGTACCTATAACC | 547 | 57 | [5] |
|  |  | R | TTGTAATTCATTAAGCATTCTGCC |  |  |  |
|  | *cmlA* | F | CCGCCACGGTGTTGTTGTTATC | 698 | 57 | [5] |
|  |  | R | CACCTTGCCTGCCCATCATTAG |  |  |  |
|  | *floR* | F | TATCTCCCTGTCGTTCCAG | 399 | 52 | [5] |
|  |  | R | AGAACTCGCCGATCAATG |  |  |  |
|  | *tetA* | F | GCTACATCCTGCTTGCCTTC | 210 | 58 | [5] |
|  |  | R | CATAGATCGCCGTGAAGAG |  |  |  |
|  | *tetB* | F | TTGGTTAGGGGCAAGTTTTG | 659 | 56 | [5] |
|  |  | R | GTAATGGGCCAATAACACCG |  |  |  |
|  | *tetD* | F | AAACCATTACGGCATTCTGC | 787 | 60 | [5] |
|  |  | R | GACCGGATACACCATCCATC |  |  |  |
|  | *qnrA* | F | ATTTCTCA CGCCAGGATTTG | 516 | 53 | [6] |
|  |  | R | GATCGGCAAAGGTTAGGTCA |  |  |  |
|  | *qnrB* | F | GATCGTGAAAGCCAGAAAGG | 469 | 53 | [6] |
|  |  | R | ACGATGCCTGGTAGTTGTCC |  |  |  |
|  | *qnrC* | F | GGGTTGTACATTTATTGAATC | 447 | 50 | [6] |
|  |  | R | TCCACTTTACGAGGTTCT |  |  |  |
|  | *qnrS1* | F | ACGACATTCGTCAACTGCAA | 417 | 53 | [6] |
|  |  | R | TAAATTGGCACCCTGTAGGC |  |  |  |
|  | *qnrS2* | F | TGGAAACCTACCGTCACACA | 600 | 60 | [7] |
|  |  | R | CTGGCAATTTTGATACCTGA |  |  |  |
|  | *aac(6)-Ib-cr* | F | TTGCGATGCTCTATGAGTGGCTA | 482 | 50 | [6] |
|  |  | R | CTCGAATGCCTGGCGTGTTT |  |  |  |
|  | *aac(3)-I* | F | ACCTACTCCCAACATCAGCC | 169 | 60 | [5] |
|  |  | R | ATATAGATCTCACTACGCGC |  |  |  |
|  | *aac(3)-II* | F | ACTGTGATGGGATACGCGTC | 237 | 60 | [5] |
|  |  | R | CTCCGTCAGCGTTTCAGCTA |  |  |  |
|  | *aac(3)-IV* | F | CTTCAGGATGGCAAGTTGGT | 286 | 60 | [5] |
|  |  | R | TCATCTCGTTCTCCGCTCAT |  |  |  |
|  | *sul1* | F | TGGTGACGGTGTTCGGCATTC | 789 | 60 | [5] |
|  |  | R | GCGAGGGTTTCCGAGAAGGTG |  |  |  |
|  | *sul2* | F | CGGCATCGTCAACATAACC | 722 | 55 | [5] |
|  |  | R | GTGTGCGGATGAAGTCAG |  |  |  |
|  | *dfrIa* | F | GTGAAACTATCACTAATGG | 474 | 55 | [5] |
|  |  | R | TTAACCCTTTTGCCAGATTT |  |  |  |
|  | *dfrIb* | F | GAGCAGCTICTITTIAAAGC | 393 | 60 | [5] |
|  |  | R | TTAGCCCTTTIICCAATTTT |  |  |  |
|  | *dfrII* | F | GATCACGTGCGCAAGAAATC | 141 | 50 | [5] |
|  |  | R | AAGCGCAGCCACAGGATAAAT |  |  |  |
|  | *dfrVII* | F | TTGAAAATTTCATTGATT | 474 | 55 | [5] |
|  |  | R | TTAGCCTTTTTTCCAAATCT |  |  |  |
|  | *dfrXII* | F | GGTGSGCAGAAGATTTTTCGC | 319 | 60 | [5] |
|  |  | R | TGGGAAGAAGGCGTCACCCTC |  |  |  |
| Replicon types | IncHI1 | F | GGAGCGATGGATTACTTCAGTAC | 471 | 60 | [8] |
|  |  | R | TGCCGTTTCACCTCGTGAGTA |  |  |  |
|  | IncHI2 | F | TTTCTCCTGAGTCACCTGTTAACAC | 644 | 60 | [8] |
|  |  | R | GGCTCACTACCGTTGTCATCCT |  |  |  |
|  | IncI1-Iγ | F | CGAAAGCCGGACGGCAGAA | 139 | 60 | [8] |
|  |  | R | TCGTCGTTCCGCCAAGTTCGT |  |  |  |
|  | IncI2 | F | CTGTCGGCATGTCTGTCTC | 553 | 55 | [9] |
|  |  | R | CTGGCTACCAGTTGCTCTAA |  |  |  |
|  | IncX1 | F | GCTTAGACTTTGTTTTATCGTT | 461 | 62 | [10] |
|  |  | R | TAATGATCCTCAGCATGTGAT |  |  |  |
|  | IncX2 | F | GCGAAGAAATCAAAGAAGCTA | 678 | 63 | [10] |
|  |  | R | TGTTGAATGCCGTTCTTGTCCAG |  |  |  |
|  | IncX3 | F | GTTTTCTCCACGCCCTTGTTCA | 351 | 63 | [10] |
|  |  | R | CTTTGTGCTTGGCTATCATAA |  |  |  |
|  | IncX4 | F | AGCAAACAGGGAAAGGAGAAGACT | 569 | 62 | [10] |
|  |  | R | TACCCCAAATCGTAACCTG |  |  |  |
|  | IncL/M | F | GGATGAAAACTATCAGCATCTGAAG | 785 | 60 | [8] |
|  |  | R | CTGCAGGGGCGATTCTTTAGG |  |  |  |
|  | IncFIA | F | CCATGCTGGTTCTAGAGAAGGTG | 462 | 60 | [8] |
|  |  | R | GTATATCCTTACTGGCTTCCGCAG |  |  |  |
|  | IncFIB | F | GGAGTTCTGACACACGATTTTCTG | 702 | 63 | [8] |
|  |  | R | CTCCCGTCGCTTCAGGGCATT |  |  |  |
|  | IncFIC | F | GTGAACTGGCAGATGAGGAAGG | 262 | 60 | [8] |
|  |  | R | TTCTCCTCGTCGCCAAACTAGAT |  |  |  |
|  | IncFIIs | F | CTGTCGTAAGCTGATGGC | 270 | 60 | [8] |
|  |  | R | CTCTGCCACAAACTTCAGC |  |  |  |
|  | IncA/C | F | GAGAACCAAAGACAAAGACCTGGA | 465 | 60 | [8] |
|  |  | R | ACGACAAACCTGAATTGCCTCCTT |  |  |  |
|  | IncP | F | CTATGGCCCTGCAAACGCGCCAGAAA | 534 | 60 | [8] |
|  |  | R | TCACGCGCCAGGGCGCAGCC |  |  |  |
|  | IncK | F | GCGGTCCGGAAAGCCAGAAAAC | 160 | 60 | [8] |
|  |  | R | TCTTTCACGAGCCCGCCAAA |  |  |  |
|  | IncB/O | F | GCGGTCCGGAAAGCCAGAAAAC | 159 | 60 | [8] |
|  |  | R | TCTGCGTTCCGCCAAGTTCGA |  |  |  |
|  | IncR | F | TCGCTTCATTCCTGCTTCAGC | 251 | 60 | [11] |
|  |  | R | GTGTGCTGTGGTTATGCCTCA |  |  |  |
|  | IncFII | F | CACACCATCCTGCACTTA | 260 | 60 | [8] |
|  |  | R | CTGATCGTTTAAGGAATTTT |  |  |  |
|  | IncN | F | GTCTAACGAGCTTACCGAAG | 559 | 55 | [8] |
|  |  | R | GTTTCAACTCTGCCAAGTTC |  |  |  |

**Supplementary Table 3. Oligonucleotide sequences and annealing temperature of primers used for virulence factor typing**

| Function | Genes |  | Nucleotide sequence | Amplicon size (bp) | Annealing temp. (℃) | Reference |
| --- | --- | --- | --- | --- | --- | --- |
| Adhesion | *fimH* | F | CTGGTCATTCGCCTGTAAAACCGCCA | 846 | 63 | [12] |
|  |  | R | GTCACGCCAATAATCGATTGCACATTCCCT |  |  |  |
|  | *iha* | F | CTGGCGGAGGCTCTGAGATCA | 827 | 55 | [13] |
|  |  | R | TCCTTAAGCTCCCGCGGCTGA |  |  |  |
|  | *papC* | F | GTGGCAGTATGAGTAATGACCGTTA | 200 | 63 | [14] |
|  |  | R | ATATCCTTTCTGCAGGGATGCAATA |  |  |  |
|  | *csgA* | F | ACTCTGACTTGACTATTACC | 200 | 55 | [12] |
|  |  | R | AGATGCAGTCTGGTCAAC |  |  |  |
|  | *sfa/focDE* | F | CTCCGGAGAACTGGGTGCATCTTAC | 410 | 60 | [15] |
|  |  | R | CGGAGGAGTAATTACAAACCTGGCA |  |  |  |
|  | *afa/draBC* | F | GCTGGGCAGCAAACTGATAACTCTC | 794 | 60 | [15] |
|  |  | R | CATCAAGCTGTTTGTTCGTCCGCCG |  |  |  |
|  | *papAH* | F | ATGGCAGTGGTGTCTTTTGGTG | 717 | 60 | [16] |
|  |  | R | CGTCCCACCATACGTGCTCTTC |  |  |  |
| Toxin | *yfcV* | F | ACATGGAGACCACGTTCACC | 292 | 60 | [17] |
|  |  | R | GTAATCTGGAATGTGGTCAGG |  |  |  |
|  | *astA* | F | TGCCATCAACACAGTATATCCG | 102 | 65 | [18] |
|  |  | R | ACGGCTTTGTAGTCCTTCCAT |  |  |  |
|  | *hlyF* | F | GGCCACAGTCGTTTAGGGTGCTTACC | 450 | 60 | [13] |
|  |  | R | GGCGGTTTAGGCATTCCGATACTCAG |  |  |  |
|  | *aat* | F | TCGGCTTATGAAGCAAAAATG | 828 | 53 | [19] |
|  |  | R | GATAACGTCGTCTTGTCCATTC |  |  |  |
|  | *pic* | F | AGCCGTTTCCGCAGAAGCC | 1111 | 63 | [18] |
|  |  | R | AAATGTCAGTGAACCGACGATTGG |  |  |  |
|  | *vat* | F | TCAGGACACGTTCAGGCATTCAGT | 1100 | 60 | [17] |
|  |  | R | GGCCAGAACATTTGCTCCCTTGTT |  |  |  |
|  | *stx1* | F | CGATGTTACGGTTTGTTACTGTGACAGC | 244 | 63 | [18] |
|  |  | R | AATGCCACGCTTCCCAGAATTG |  |  |  |
|  | *stx2* | F | GTTTTGACCATCTTCGTCTGATTATTGAG | 324 | 63 | [18] |
|  |  | R | AGCGTAAGGCTTCTGCTGTGAC |  |  |  |
|  | *aggR* | F | ACGCAGAGTTGCCTGATAAAG | 400 | 63 | [18] |
|  |  | R | AATACAGAATCGTCAGCATCAGC |  |  |  |
|  | *elt* | F | GAACAGGAGGTTTCTGCGTTAGGTG | 655 | 63 | [18] |
|  |  | R | CTTTCAATGGCTTTTTTTTGGGAGTC |  |  |  |
|  | *est1b* | F | TGTCTTTTTCACCTTTCGCTC | 171 | 63 | [18] |
|  |  | R | CGGTACAAGCAGGATTACAACAC |  |  |  |
|  | *est1a* | F | CCTCTTTTAGYCAGACARCTGAATCASTTG | 157 | 63 | [18] |
|  |  | R | CAGGCAGGATTACAACAAAGTTCACAG |  |  |  |
|  | *bfpB* | F | GACACCTCATTGCTGAAGTCG | 910 | 63 | [18] |
|  |  | R | CCAGAACACCTCCGTTATGC |  |  |  |
|  | *eaeA* | F | TCAATGCAGTTCCGTTATCAGTT | 482 | 63 | [18] |
|  |  | R | GTAAAGTCCGTTACCCCAACCTG |  |  |  |
|  | *invE* | F | CGATAGATGGCGAGAAATTATATCCCG | 766 | 63 | [18] |
|  |  | R | CGATCAAGAATCCCTAACAGAAGAATCAC |  |  |  |
| Protectin | *traT* | F | GGTGTGGTGCGATGAGCACAG | 290 | 60 | [12] |
|  |  | R | CACGGTTCAGCCATCCCTGAG |  |  |  |
|  | *ompT* | F | TCATCCCGGAAGCCTCCCTCACTACTAT | 496 | 64 | [13] |
|  |  | R | TAGCGTTTGCTGCACTGGCTTCTGATAC |  |  |  |
|  | *kpsMTII* | F | GCGCATTTGCTGATACTGTTG | 272 | 60 | [20] |
|  |  | R | CATCCAGACGATAAGCATGAGC |  |  |  |
|  | *iss* | F | CAGCAACCCGAACCACTTGATG | 323 | 60 | [13] |
|  |  | R | AGCATTGCCAGAGCGGCAGAA |  |  |  |
| Sidero-  phores | *fyuA* | F | TGATTAACCCCGCGACGGGAA | 880 | 63 | [12] |
|  |  | R | CGCAGTAGGCACGATGTTGTA |  |  |  |
|  | *iroNe.coli* | F | AAGTCAAAGCAGGGGTTGCCCG | 665 | 63 | [13] |
|  |  | R | GACGCCGACATTAAGACGCAG |  |  |  |
|  | *iutA* | F | GGCTGGACATCATGGGAACTGG | 302 | 60 | [13] |
|  |  | R | CGTCGGGAACGGGTAGAATCG |  |  |  |
|  | *chuA* | F | CTGAAACCATGACCGTTACG | 652 | 55 | [4] |
|  |  | R | TTGTAGTAACGCACTAAACC |  |  |  |
| Phylogenetic group | *yjaA* | F | CAAACGTGAAGTGTCAGGAG | 288 | 55 | [4] |
|  |  | R | AATGCGTTCCTCAACCTGTG |  |  |  |
|  | *chuA* | F | ATGGTACCGGACGAACCAAC | 211 | 55 | [4] |
|  |  | R | TGCCGCCAGTACCAAAGACA |  |  |  |
|  | *tspE4.C2* | F | CACTATTCGTAAGGTCATCC | 152 | 55 | [4] |
|  |  | R | AGTTTATCGCTGCGGGTCGC |  |  |  |
|  | *AceK.f* | F | AACGCTATTCGCCAGCTTGC | 400 | 55 | [4] |
|  | *ArpA1.r* | R | TCTCCCCATACCGTACGCTA |  |  |  |
|  | *ArpAgpE.f* | F | GATTCCATCTTGTCAAAATATGCC | 301 | 55 | [4] |
|  | *ArpAgpE.r* | R | GAAAAGAAAAAGAATTCCCAAGAG |  |  |  |
|  | *trpAgpC.1* | F | AGTTTTATGCCCAGTGCGAG | 219 | 55 | [4] |
|  | *trpAgpC.2* | R | TCTGCGCCGGTCACGCCC |  |  |  |
|  | *trpBA.f* | F | CGGCGATAAAGACATCTTCAC | 489 | 55 | [4] |
|  | *trpBA.r* | R | GCAACGCGGCCTGGCGGAAG |  |  |  |

**Supplementary Table 4. Antimicrobial susceptibility of MCR1-EC isolates according to pathogenic *E. coli* types**

| Antimicrobial Classes | Antimicrobial  Agents | Total  (n=53) | Commensal-EC  (n=37) | InPEC  (n=12) | ExPEC  (n=4) |
| --- | --- | --- | --- | --- | --- |
| Broad-spectrum  penicillin | ampicillin | 84.9% | 83.8% | 83.3% | 100.0% |
| 3rd-generation  Cephalosporin | cefotaxime | 17.0% | 16.2% | 0.0% | 75.0% |
|  | ceftazidime | 3.8% | 5.4% | 0.0% | 0.0% |
|  | ceftriaxone | 17.0% | 16.2% | 0.0% | 75.0% |
| β-lactamase inhibitor | amoxicillin/clavulanate | 13.2% | 13.5% | 8.3% | 25.0% |
| Monobactam | aztreonam | 11.3% | 10.8% | 0.0% | 50.0% |
| Carbapenem | imipenem | 0.0% | 0.0% | 0.0% | 0.0% |
| Phenicol | chloramphenicol | 67.9% | 78.4% | 25.0% | 100.0% |
| Aminoglycoside | amikacin | 0.0% | 0.0% | 0.0% | 0.0% |
|  | gentamycin | 15.1% | 13.5% | 16.7% | 25.0% |
| Tetracycline | tetracycline | 88.7% | 83.8% | 100.0% | 100.0% |
| Quinolone | nalidixic acid | 39.6% | 45.9% | 0.0% | 100.0% |
|  | ciprofloxacin | 32.1% | 35.1% | 0.0% | 100.0% |
| Sulfonamide/  Trimethoprim | sulfamethoxazole/  trimethoprim | 37.7% | 29.7% | 66.7% | 25.0% |
| Average number of classes to which strains are resistant  (max=11) | - | 4.8 | 4.8 | 4.0 | 7.0 |

*Abbreviation. Commensal-EC, Commensal *E. coli*; InPEC, Intestinal pathogenic *E. coli*; ExPEC, Extra-intestinal pathogenic *E. coli*

**Supplementary Table 5. Prevalence of antimicrobial resistance genes and their correlation coefficients with expected phenotypic resistance of MCR1-EC isolates**

| Antimicrobial Classes | Antimicrobial  Resistance Gene | Prevalence (%) | No. of positive MCR1-EC  /No. of total MCR1-EC | ^a^Correlation Coefficient | *Ρ* - value |
| --- | --- | --- | --- | --- | --- |
| Narrow-spectrum  β-lactams | *bla_TEM-1_* | 47.2 | 25/53 | 0.398 | <0.01^*^ |
|  | *bla_TEM-215_* | 5.7 | 3/53 | 0.103 | 0.46 |
|  | *bla_TEM-237_* | 3.8 | 2/53 | 0.083 | 0.55 |
|  | *bla_TEM-20_* | 1.9 | 1/53 | 0.058 | 0.68 |
|  | *bla_TEM-1,215,237, or 20_* | 58.8 | 31/53 | 0.377 | <0.01^*^ |
| Extra-spectrum  β-lactams | *bla_CTX-M-55_* | 17.1 | 9/53 | 0.932 | <0.01^*^ |
| Phenicol | *catA* | 3.8 | 2/53 | 0.142 | 0.31 |
|  | *cmlA* | 3.8 | 2/53 | 0.142 | 0.31 |
|  | *floR* | 69.8 | 37/53 | 0.917 | <0.01^*^ |
|  | *catA, cmlA, or floR* | 69.8 | 37/53 | 0.917 | <0.01^*^ |
| Aminoglycoside | *aac(3)-I* | 0.0 | 0/53 | - | - |
|  | *aac(3)-II* | 7.5 | 4/53 | 0.478 | <0.01^*^ |
|  | *aac(3)-IV* | 1.9 | 1/53 | 0.329 | 0.02^*^ |
|  | *aac-(3)-I, II, or IV* | 9.4 | 5/53 | 0.585 | <0.01^*^ |
| Tetracycline | *tetA* | 79.2 | 42/53 | 0.350 | 0.01^*^ |
|  | *tetB* | 11.3 | 6/53 | 0.139 | 0.32 |
|  | *tetD* | 0.0 | 0/53 | - | - |
|  | *tetA, B, or D* | 90.6 | 48/53 | 0.637 | <0.01^*^ |
| Quinolone | *qnrA* | 0.0 | 0/53 | - | - |
|  | *qnrB* | 0.0 | 0/53 | - | - |
|  | *qnrC* | 0.0 | 0/53 | - | - |
|  | *qnrS1* | 41.5 | 22/53 | -0.213 | 0.13 |
|  | *qnrS2* | 37.7 | 20/53 | -0.074 | 0.60 |
|  | *aac(6)-cr-Ib* | 0.0 | 0/53 | - | - |
|  | *qnrA, B, C, S1, S2*  *or aac(6)-cr-Ib* | 45.3 | 24/53 | -0.117 | 0.40 |
| Sulfonamide/  Trimethoprim | *sul1* | 11.3 | 6/53 | 0.336 | 0.01^*^ |
|  | *sul2* | 50.9 | 27/53 | 0.608 | <0.01^*^ |
|  | *dfrIa* | 26.4 | 14/53 | 0.593 | <0.01^*^ |
|  | *dfrIb* | 0.0 | 0/53 | - | - |
|  | *dfrII* | 0.0 | 0/53 | - | - |
|  | *dfrVII* | 0.0 | 0/53 | - | - |
|  | *dfrXII* | 9.4 | 5/53 | 0.415 | <0.01^*^ |
|  | *sul1, sul2, dfrIa, Ib, II, VII, or XII* | 56.6 | 30/53 | 0.603 | <0.01^*^ |

^a^ Correlation coefficience of the carriage of antimicrobial resistance genes with expected phenotypic resistance was calculated via Spearman’s correlation test

^*^*Ρ* < 0.05, statistically significant

**Supplementary Table 6. Prevalence of antimicrobial resistance genes and their correlation coefficients with expected phenotypic resistance of MCR1-EC isolates**

| Replicon types | Prevalence (%) | No. of positive MCR1-EC  /No. of total MCR1-EC |
| --- | --- | --- |
| IncI2 | 94.3 | 50/53 |
| IncFIB | 84.9 | 45/53 |
| IncFII | 67.9 | 36/53 |
| IncFIC | 43.4 | 23/53 |
| IncR | 28.3 | 15/53 |
| IncI1-Iγ | 28.3 | 15/53 |
| IncX1 | 17.0 | 9/53 |
| IncX4 | 7.5 | 4/53 |
| IncFIA | 7.5 | 4/53 |
| IncN | 1.9 | 1/53 |
| IncB/O | 1.9 | 1/53 |
| IncHI1 | 0.0 | 0/53 |
| IncHI2 | 0.0 | 0/53 |
| IncA/C | 0.0 | 0/53 |

**Supplementary Table 7. Prevalence of virulence factor classes according to the four swine production stages**

^a^ Where zeros cause problems in calculating OR or 95% CI, Fisher’s exact test was used in the calculations instead of GEE.

^*^*Ρ* < 0.05, statistically significant based on GEE

^†^*Ρ* < 0.05, statistically significant based on Fisher’s exact test

| Virulence Factor classes | Weaning pigs  (Reference) | | |  | Growing pigs | | |  | Finishing pigs | | |  | Pregnant sows | | |
| --- | --- | --- | --- | --- | --- | --- | --- | --- | --- | --- | --- | --- | --- | --- | --- |
|  | Prevalence  (%) | OR  (95% CI) | *Ρ -* value |  | Prevalence  (%) | OR  (95% CI) | *Ρ -* value |  | Prevalence  (%) | OR  (95% CI) | *Ρ -* value |  | Prevalence  (%) | OR  (95% CI) | *Ρ -* value |
| Adhesion | 100.0 | - | - |  | 100.0 | 1.3 ^a^  (003-71.86) | 0.88 |  | 100.0 | 0.9 ^a^  (0.02-46.71) | 0.94 |  | 100.0 | 0.6 ^a^  (0.01-32.34) | 0.79 |
| Toxin | 35.7 | - | - |  | 31.6 | 1.2  (0.11-13.77) | 0.88 |  | 33.3 | 1.1  (0.18-6.79) | 0.91 |  | 25.0 | 1.7  (0.50-5.54) | 0.41 |
| Protectin | 85.7 | - | - |  | 100.0 | 7.8 ^a^  (0.35-176.35) | 0.20 |  | 91.7 | 1.8 ^a^  (0.15-23.16) | 0.64 |  | 75.0 | 0.5 ^a^  (0.06-4.47) | 0.54 |
| Sidero-phore | 42.9 | - | - |  | 31.6 | 0.62  (0.04-8.75) | 0.72 |  | 16.7 | 0.27  (0.04-2.02) | 0.20 |  | 62.5 | 2.2  (0.57-8.67) | 0.25 |

**References**

[1] Lee S, An JU, Guk JH, Song H, Yi S, Kim WH, et al. Prevalence, Characteristics and Clonal Distribution of Extended-Spectrum beta-Lactamase- and AmpC beta-Lactamase-Producing Escherichia coli Following the Swine Production Stages, and Potential Risks to Humans. Front Microbiol. 2021;12:710747.

[2] Wu R, Yi LX, Yu LF, Wang J, Liu Y, Chen X, et al. Fitness Advantage of mcr-1-Bearing IncI2 and IncX4 Plasmids in Vitro. Front Microbiol. 2018;9:331.

[3] Jouini A, Vinue L, Slama KB, Saenz Y, Klibi N, Hammami S, et al. Characterization of CTX-M and SHV extended-spectrum beta-lactamases and associated resistance genes in Escherichia coli strains of food samples in Tunisia. J Antimicrob Chemother. 2007;60:1137-41.

[4] Clermont O, Christenson JK, Denamur E, Gordon DM. The Clermont Escherichia coli phylo-typing method revisited: improvement of specificity and detection of new phylo-groups. Environ Microbiol Rep. 2013;5:58-65.

[5] Saenz Y, Brinas L, Dominguez E, Ruiz J, Zarazaga M, Vila J, et al. Mechanisms of resistance in multiple-antibiotic-resistant Escherichia coli strains of human, animal, and food origins. Antimicrob Agents Chemother. 2004;48:3996-4001.

[6] Liao XP, Xia J, Yang L, Li L, Sun J, Liu YH, et al. Characterization of CTX-M-14-producing Escherichia coli from food-producing animals. Front Microbiol. 2015;6:1136.

[7] Arias A, Seral C, Navarro F, Miro E, Coll P, Castillo FJ. Plasmid-mediated QnrS2 determinant in an Aeromonas caviae isolate recovered from a patient with diarrhoea. Clin Microbiol Infect. 2010;16:1005-7.

[8] Carattoli A, Bertini A, Villa L, Falbo V, Hopkins KL, Threlfall EJ. Identification of plasmids by PCR-based replicon typing. J Microbiol Methods. 2005;63:219-28.

[9] Lv L, Partridge SR, He L, Zeng Z, He D, Ye J, et al. Genetic characterization of IncI2 plasmids carrying blaCTX-M-55 spreading in both pets and food animals in China. Antimicrob Agents Chemother. 2013;57:2824-7.

[10] Johnson TJ, Bielak EM, Fortini D, Hansen LH, Hasman H, Debroy C, et al. Expansion of the IncX plasmid family for improved identification and typing of novel plasmids in drug-resistant Enterobacteriaceae. Plasmid. 2012;68:43-50.

[11] Garcia-Fernandez A, Fortini D, Veldman K, Mevius D, Carattoli A. Characterization of plasmids harbouring qnrS1, qnrB2 and qnrB19 genes in Salmonella. J Antimicrob Chemother. 2009;63:274-81.

[12] Gonzalez Moreno C, Torres Luque A, Oliszewski R, Rosa RJ, Otero MC. Characterization of native Escherichia coli populations from bovine vagina of healthy heifers and cows with postpartum uterine disease. PLoS One. 2020;15:e0228294.

[13] Aazam K, Hassan M, Mahbobe M. Detection of uropathogenic Escherichia coli virulence factors in patients with urinary tract infections in Iran. African Journal of Microbiology Research. 2012;6:6811-6.

[14] Zhi S, Banting G, Li Q, Edge TA, Topp E, Sokurenko M, et al. Evidence of Naturalized Stress-Tolerant Strains of Escherichia coli in Municipal Wastewater Treatment Plants. Appl Environ Microbiol. 2016;82:5505-18.

[15] Le Bouguenec C, Archambaud M, Labigne A. Rapid and specific detection of the pap, afa, and sfa adhesin-encoding operons in uropathogenic Escherichia coli strains by polymerase chain reaction. J Clin Microbiol. 1992;30:1189-93.

[16] Johnson JR, Porter S, Johnston B, Kuskowski MA, Spurbeck RR, Mobley HL, et al. Host Characteristics and Bacterial Traits Predict Experimental Virulence for Escherichia coli Bloodstream Isolates From Patients With Urosepsis. Open Forum Infect Dis. 2015;2:ofv083.

[17] Spurbeck RR, Dinh PC, Jr., Walk ST, Stapleton AE, Hooton TM, Nolan LK, et al. Escherichia coli isolates that carry vat, fyuA, chuA, and yfcV efficiently colonize the urinary tract. Infect Immun. 2012;80:4115-22.

[18] Muller D, Greune L, Heusipp G, Karch H, Fruth A, Tschape H, et al. Identification of unconventional intestinal pathogenic Escherichia coli isolates expressing intermediate virulence factor profiles by using a novel single-step multiplex PCR. Appl Environ Microbiol. 2007;73:3380-90.

[19] Moyo SJ, Maselle SY, Matee MI, Langeland N, Mylvaganam H. Identification of diarrheagenic Escherichia coli isolated from infants and children in Dar es Salaam, Tanzania. BMC Infect Dis. 2007;7:92.

[20] Johnson JR, Stell AL. Extended virulence genotypes of Escherichia coli strains from patients with urosepsis in relation to phylogeny and host compromise. J Infect Dis. 2000;181:261-72.
